# Modeling Electrophysiological Coupling and Fusion between Human Mesenchymal Stem Cells and Cardiomyocytes

**DOI:** 10.1371/journal.pcbi.1005014

**Published:** 2016-07-25

**Authors:** Joshua Mayourian, Ruben M. Savizky, Eric A. Sobie, Kevin D. Costa

**Affiliations:** 1 Cardiovascular Research Center, Icahn School of Medicine at Mount Sinai, New York, New York, United States of America; 2 Department of Chemistry, The Cooper Union, New York, New York, United States of America; 3 Department of Pharmacology and Systems Therapeutics, Icahn School of Medicine at Mount Sinai, New York, New York, United States of America; University of Virginia, UNITED STATES

## Abstract

Human mesenchymal stem cell (hMSC) delivery has demonstrated promise in preclinical and clinical trials for myocardial infarction therapy; however, broad acceptance is hindered by limited understanding of hMSC-human cardiomyocyte (hCM) interactions. To better understand the electrophysiological consequences of direct heterocellular connections between hMSCs and hCMs, three original mathematical models were developed, representing an experimentally verified triad of hMSC families with distinct functional ion channel currents. The arrhythmogenic risk of such direct electrical interactions in the setting of healthy adult myocardium was predicted by coupling and fusing these hMSC models to the published ten Tusscher midcardial hCM model. Substantial variations in action potential waveform—such as decreased action potential duration (APD) and plateau height—were found when hCMs were coupled to the two hMSC models expressing functional delayed rectifier-like human ether à-go-go K^+^ channel 1 (hEAG1); the effects were exacerbated for fused hMSC-hCM hybrid cells. The third family of hMSCs (Type C), absent of hEAG1 activity, led to smaller single-cell action potential alterations during coupling and fusion, translating to longer tissue-level mean action potential wavelength. In a simulated 2-D monolayer of cardiac tissue, re-entry vulnerability with low (5%) hMSC insertion was approximately eight-fold lower with Type C hMSCs compared to hEAG1-functional hMSCs. A 20% decrease in APD dispersion by Type C hMSCs compared to hEAG1-active hMSCs supports the claim of reduced arrhythmogenic potential of this cell type with low hMSC insertion. However, at moderate (15%) and high (25%) hMSC insertion, the vulnerable window increased independent of hMSC type. In summary, this study provides novel electrophysiological models of hMSCs, predicts possible arrhythmogenic effects of hMSCs when directly coupled to healthy hCMs, and proposes that isolating a subset of hMSCs absent of hEAG1 activity may offer increased safety as a cell delivery cardiotherapy at low levels of hMSC-hCM coupling.

## Author Summary

Myocardial infarction—better known as a heart attack—strikes on average every 43 seconds in America. An emerging approach to treat myocardial infarction patients involves the delivery of human mesenchymal stem cells (hMSCs) to the damaged heart. While clinical trials of this therapeutic approach have yet to report adverse effects on heart electrical rhythm, such consequences have been implicated in simpler experimental systems and thus remain a concern. In this study, we utilized mathematical modeling to simulate electrical interactions arising from direct coupling between hMSCs and human heart cells to develop insight into the possible adverse effects of this therapeutic approach on human heart electrical activity, and to assess a novel strategy for reducing some potential risks of this therapy. We developed the first mathematical models of electrical activity of three families of hMSCs based on published experimental data, and integrated these with previously established mathematical models of human heart cell electrical activity. Our computer simulations demonstrated that one particular family of hMSCs minimized the disturbances in cardiac electrical activity both at the single-cell and tissue levels, suggesting that isolating this specific sub-population of hMSCs for myocardial delivery could potentially increase the safety of future hMSC-based heart therapies.

## Introduction

Ischemic heart disease, which results from reduced coronary flow of oxygenated blood, is a leading cause of myocardial infarction and heart failure. This insufficient oxygenation results in the death of cardiomyocytes, which are normally incapable of substantial regeneration. Therefore, despite tremendous advancements in pharmacological and interventional therapeutic approaches, ischemic heart disease continues to be responsible for nearly 1 out of 6 deaths in the United States [1, 2]. This has motivated novel cardiotherapeutic strategies to repair and regenerate heart muscle, including human mesenchymal stem cell (hMSC) therapy, the method of interest in this study.

In clinical trials for treating myocardial infarction, the delivery of autologous bone marrow derived hMSCs has demonstrated improved ventricular ejection, enhanced angiogenesis, decreased fibrosis and scar size, and minimal immune response [3]. However, the benefits have often been modest and transient [4, 5], underscoring a need to better understand and exploit the underlying mechanisms by which hMSCs interact with human cardiomyocytes (hCMs) [6]. This limited mechanistic knowledge further makes it difficult to ensure long-term stability, with seamless structural and functional integration into the host tissue [7–9]. Therefore, deeper investigation into the mechanisms of how hMSCs impact cardiac function is necessary.

Proposed hMSC-hCM interactions predominantly include: reprogramming of host hCMs, transdifferentiation of hMSCs into hCMs, paracrine signaling, electrophysiological coupling, and cellular fusion [6, 10]. Indirect paracrine signaling through the release of largely unidentified soluble factors is thought to play an important role [6, 11]; however, hMSCs have also exhibited functional direct electrical interactions with cardiomyocytes both in vitro and in vivo [10, 12–17], motivating ongoing investigations of the electrophysiological coupling and cellular fusion mechanisms. In particular, Valiunas et al. showed that hMSCs form connexin 43-mediated gap junctions between each other and with acutely isolated canine cardiomyocytes, suggesting the ability to form heterocellular electrical networks [15]. Later in vitro studies showed that such electrical connections can be functional and potentially arrhythmogenic, as co-culturing murine cardiomyocytes with greater than 10 percent of hMSCs decreased conduction velocity (CV) and predisposed re-entrant arrhythmias [16]. Pro-arrhythmic characteristics were also detected in vivo, where pigs receiving intravenous injections of mesenchymal stem cells had decreased effective refractory periods [17]. Moreover, Shadrin et al. recently reported a 25–40% incidence of hybrid cell formation of hMSCs and neonatal rat ventricular myocytes through cell fusion [10]. However, species-specific effects can limit the clinical relevance of such animal and in vitro studies, and similarly controlled experiments are difficult to perform in human patients. While hMSC therapy clinical trials are yet to report arrhythmogenicity [18], such adverse effects remain a concern. Therefore, in this study, it was of interest to assess the electrophysiological safety of various levels of direct hMSC-hCM electrical interactions under healthy conditions [18], and to predict methods of improving the safety of this therapy.

Mathematical modeling is a powerful tool that can simulate direct intercellular electrical interactions between hMSCs and hCMs. Electrophysiological models have been established to describe hCMs [19–21], as well as their interactions with other resident heart cells [22–25], but never before with hMSCs. Therefore, in this study, the various types of currents experimentally characterized in hMSCs [26–29] were mathematically modeled to simulate an empirically classified triad of hMSC families distinguished by their respective functional ion channels: Type A) delayed rectifier-like hEAG1 and calcium activated potassium currents; Type B) delayed rectifier-like hEAG1, calcium activated potassium, tetrodotoxin (TTX)-sensitive sodium, and L-type calcium currents; and Type C) calcium activated potassium and transient outward currents [26, 28]. The empirical distinction of these three hMSC families was originally reported by Li et al. [26] based on patch clamp measurements of bone marrow-derived hMSCs obtained commercially and maintained in monolayer culture. We then simulated the electrical activity of hMSCs coupled to healthy hCMs, and interpreted the model findings within the context of prior in vitro and in vivo experiments to identify possible opportunities to minimize arrhythmic potential in future hMSC-based cell delivery cardiotherapies.

## Methods

### hMSC Model Development

The hMSC transmembrane voltage can be modeled as:

$$\begin{array}{c} \frac{dV}{dt}=-\frac{1}{C_{m}}\left(I_{\text{stim}}+I_{\text{tot},i}\right) \end{array}$$ (1)

where *V* is voltage, *t* is time, *C*_m_ is the cell capacitance, *I*_stim_ is a stimulus current, and *I*_tot,*i*_ is the total transmembrane ionic current of Type *i* hMSCs (where *i* = A, B, or C). The total transmembrane ionic current for Types A, B, and C hMSCs are given by Eqs 2, 3 and 4, respectively:

$$\begin{array}{c} I_{\text{tot},A}=I_{\text{KCa}}+I_{\text{dr}}+I_{L,A} \end{array}$$ (2)

$$\begin{array}{c} I_{\text{tot},B}=I_{\text{KCa}}+I_{\text{dr}}+I_{\text{LCa}}+I_{\text{Na}}+I_{L,B} \end{array}$$ (3)

$$\begin{array}{c} I_{\text{tot},C}=I_{\text{KCa}}+I_{\text{to}}+I_{L,C} \end{array}$$ (4)

where *I*_KCa_ is the calcium activated potassium current, *I*_dr_ is the delayed rectifier-like hEAG1 current, *I*_L,*i*_ is the leakage current for hMSC Type *i* (where *i* = A, B, or C), *I*_LCa_ is the L-type calcium current, *I*_Na_ is the TTX-sensitive sodium current, and *I*_to_ is the transient outward current.

### Modeling hMSC Ionic Currents

To describe each type of hMSC ionic current, either Hodgkin-Huxley-like or Markovian-like approaches were taken. Parameters for these models were fit to published experimental hMSC data using numerical methods described in S1 Text. Parameters used in this study can be found in Tables A-G of S1 Text.

#### Calcium activated potassium channel current

**Fig 1:**
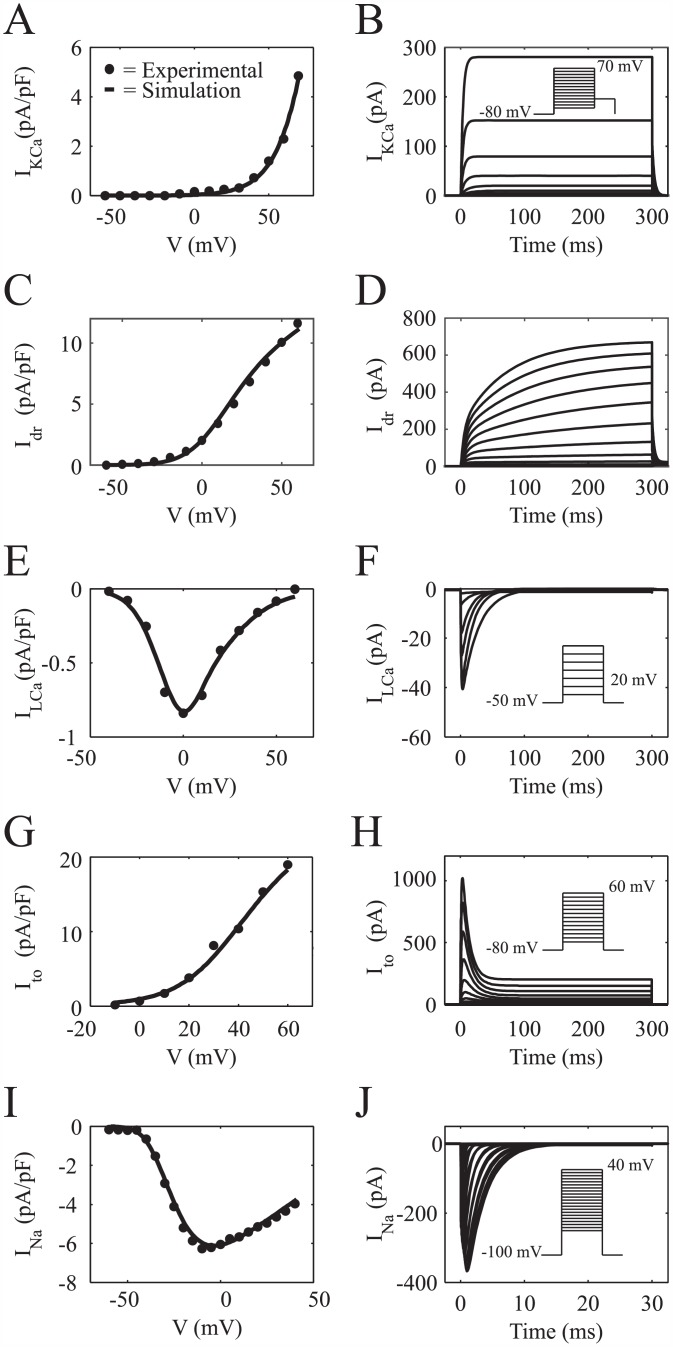
*I*-*V* and Voltage Clamp Simulations of hMSC Currents. Comparison of experimental and fitted *I*-*V* curves for hMSC channels, and the resulting voltage clamp simulations. (**A**) Fitted *I*-*V* curve for I_KCa_ together with mean experimental data from Li et al. [26]. (**B**) Simulated voltage clamp experiment of I_KCa_ (voltage step protocol inset). (**C**) Theoretical *I*-*V* curve for I_dr_ and its fit to mean experimental data [26]. (**D**) In silico voltage clamp experiment of I_dr_ (voltage step protocol shown in inset of Fig 3C). (**E**) A comparison of fitted theoretical and mean experimental *I*-*V* curve data [26] for I_LCa_. (**F**) Voltage clamp simulation for I_LCa_ (voltage step protocol inset). Comparisons between fitted theoretical and experimental *I*-*V* data [26] for I_to_ and I_Na_ are shown in (**G**) and (**I**), respectively. Voltage clamp simulations for I_to_ and I_Na_ are shown in (**H**) and (**J**), respectively (voltage step protocols inset).

To describe hMSC *I*_KCa_ encoded by the KCNMA1 gene [26], we modified the Gerstner et al. [30] persistent model, such that:

$$\begin{array}{c} I_{\text{KCa}}=G_{\text{KCa}}x\left(V-E_{K}\right) \end{array}$$ (5)

where *G*_KCa_ is the maximum conductance of the channel, *V* is the membrane potential, *E*_*K*_ is the potassium Nernst potential, and *x* is an activation variable. The voltage- and intracellular calcium concentration-dependent activation kinetics of *I*_KCa_ described by Gerstner et al. [30] was simplified by assuming constant intracellular calcium concentration. The activation parameters in this model were fit to values derived from hMSC *I*_KCa_ experimental data reported by Li et al. [26]. Fig 1A shows the simulated steady-state current-voltage (*I*-*V*) relationship together with mean experimental data. A simulation of a 300 ms voltage clamp experiment is shown in Fig 1B (voltage clamp protocol inset), demonstrating the time-dependent behavior of this current. S1 Fig shows the steady-state activation and time constant curves used in the model with corresponding values derived from experimental data [26]. The resulting formulas and fitted model parameters to fully describe *I*_KCa_ are shown in Table B of S1 Text.

#### Delayed rectifier potassium channel current

**Fig 2:**
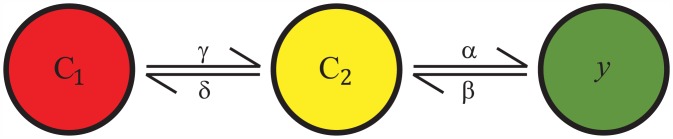
Voltage-Sensor Transition Model for the Delayed Rectifier Current. To model I_dr_, a modified version of Silverman’s two voltage-sensor transition Markovian-like model [33, 34] was used. As shown, there is one closed state (C_1_), one intermediate state (C_2_), and one open state (*y*). Each state has its own voltage-dependent rate functions (i.e., *α*, *β*, *γ*, and *δ*).

**Fig 3:**
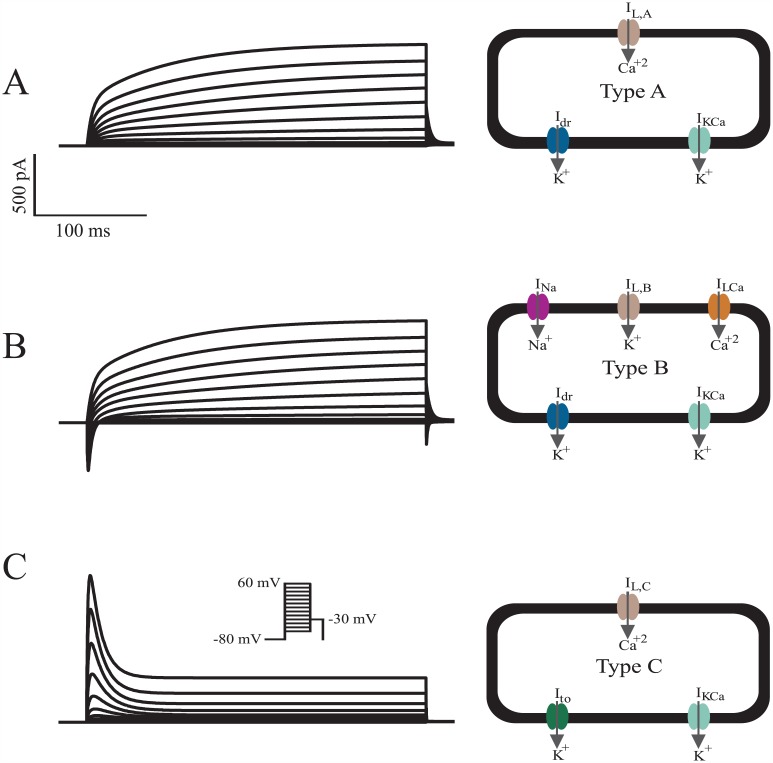
Total Current Simulations of the Triad of hMSC Families. The whole-cell models developed in this study were validated by simulating *I*_tot,A_, *I*_tot,B_, and *I*_tot,C_, as shown in (**A**), (**B**), and (**C**), respectively. Schematics of functional currents for each cell type are shown to the right of each simulation. The voltage protocol for each cell type is inset in (**C**). The simulations generally agree with the magnitude and behavior of representative experimental data [26], and thus were used to predict the direct electrical interactions between hMSCs and hCMs.

The hMSC *I*_dr_ encoded by the hEAG1 gene [26] is also persistent. Interestingly, one of its sub-families, the hEAG-related gene [31], is responsible for the rapid repolarization of the cardiac action potential [32]. To model this channel, we modified Silverman’s two voltage-sensor transition model (Fig 2) [33, 34], with one closed state (C_1_), one intermediate state (C_2_), and one open state (*y*):

$$\begin{array}{c} \frac{dC_{1}}{dt}=\delta C_{2}-\gamma C_{1} \end{array}$$ (6)

$$\begin{array}{c} \frac{dC_{2}}{dt}=\gamma C_{1}+\beta y-\left(\alpha +\delta \right)C_{2} \end{array}$$ (7)

$$\begin{array}{c} \frac{dy}{dt}=\alpha C_{2}-\beta y \end{array}$$ (8)

where *α*, *β*, *γ*, and *δ* are voltage-dependent rate functions. The resulting *I*_dr_ model is:

$$\begin{array}{c} I_{\text{dr}}=G_{\text{dr}}y\left(V-E_{k}\right) \end{array}$$ (9)

where *G*_dr_ is the maximum conductance of the channel, *y* is the activation variable, and *E*_k_ is the potassium Nernst potential. The activation parameters in this model were fit to values derived from experimental hMSC *I*_dr_ data from Li et al. [26]. The fitted *I*-*V* relationship and the corresponding mean experimental data are shown in Fig 1C. A simulation of a 300 ms voltage clamp experiment is shown in Fig 1D (voltage clamp protocol shown in Fig 3C inset), providing insight into the transient behavior of this current. S2 Fig shows the steady-state activation curve and time constant curves, in agreement with empirical results by Li et al. [26]. The resulting formulas and fitted model parameters to fully describe *I*_dr_ are shown in Table C of S1 Text.

#### L-type calcium current

The hMSC *I*_LCa_ encoded by the CACNA1C gene [26] is also found with hCMs, where it is responsible for the plateau phase of the cardiac action potential. The driving force of *I*_LCa_ was described by the Goldman-Hodgkin-Katz equation [35] to account for the large Ca^+2^ concentration gradient across the cell membrane [30, 36]. Therefore, *I*_LCa_ was defined as:

$$\begin{array}{c} I_{\text{LCa}}=G_{\text{LCa}}df4\frac{VF^{2}}{\text{RT}}\frac{\left[{\text{Ca}}_{i}^{+2}\right]e^{2VF/\text{RT}}-\phi \left[{\text{Ca}}_{o}^{+2}\right]}{e^{2VF/\text{RT}}-1} \end{array}$$ (10)

where *G*_LCa_ is the maximum conductance, *F* is Faraday’s constant, *ϕ* is the partition coefficient, and *d* and *f* are activation and inactivation gate variables, respectively. The Goldman-Hodgkin-Katz equation was simplified in this study by assuming constant intracellular and extracellular calcium concentrations. Each gate is described by Hodgkin-Huxley-type differential equations [37]. Both steady-state *d* and *f* functions, as well as their respective time constants, were fit to values derived from experimental hMSC *I*_LCa_ data from Li et al. [26]. The fitted peak *I*-*V* relationship is in agreement with mean experimental data, as shown in Fig 1E. A simulation of a 300 ms voltage clamp experiment is shown in Fig 1F (voltage clamp protocol inset), demonstrating the transient behavior of this current. S3 Fig shows the steady-state activation curve, steady-state inactivation curve, and the time constants used in the model together with values derived from experimental data [26]. The resulting formulas and fitted model parameters to fully describe *I*_LCa_ are shown in Table D of S1 Text.

#### Transient outward potassium channel current

Similar to the transient outward current of human atrial myocytes and Purkinje fiber cells [38, 39], the hMSC (*I*_to_) encoded by Kv4.2 and Kv1.4 genes displays both ephemeral and sustained currents [26]. Transient outward current is also found with hCMs, where it contributes to the notch of the cardiac action potential. To account for both the transient and sustained currents within the hMSC model, a modified version of the Nygren et al. model [40] was used:

$$\begin{array}{c} I_{\text{to}}=G_{\text{to}}rs\left(V-E_{K}\right)+G_{\text{to},\text{sus}}r_{\text{sus}}\left(V-E_{K}\right) \end{array}$$ (11)

where *G*_to_ and *G*_to,sus_ are the maximum conductances of the transient and sustained portions of the channel, respectively. *r* and *s* are activation and inactivation variables of the transient behavior, respectively, and *r*_sus_ is the activation variable of the sustained behavior. Each gate is described by Hodgkin-Huxley-type equations [37]. Steady-state functions and their respective time constants were fit to values derived from experimental hMSC *I*_to_ data from Li et al. [26]. The fitted peak *I*-*V* relationship is in agreement with experimental data, as shown in Fig 1G. A simulation of a 300 ms voltage clamp experiment is shown in Fig 1H (voltage clamp protocol inset), demonstrating the transient behavior of this current. S4 Fig shows the steady-state activation curves (*r*_∞_ and *r*_sus_), the steady-state inactivation curve, and the time constants used in the model together with values derived from experimental data [26]. The fitted formulas to fully describe *I*_to_ are shown in Table E of S1 Text.

#### TTX-sensitive sodium channel current

A modified version of the Hodgkin-Huxley two gates formula [30, 37, 41] was used to describe the hMSC *I*_Na_ encoded by the hNE-Na gene [26], such that:

$$\begin{array}{c} I_{\text{Na}}=G_{\text{Na}}m^{3}h\left(V-E_{\text{Na}}\right) \end{array}$$ (12)

where *G*_Na_ is the maximum sodium conductance, *m* is an activation gate, *h* is an inactivation gate, and *E*_Na_ is the sodium Nernst potential. Steady-state *m* and *h* functions, and their respective time constants were fit to values derived from experimental hMSC *I*_Na_ data from Li et al. [26]. The fitted peak *I*-*V* relationship is compared to mean experimental data, as shown in Fig 1I. A simulation of a 30 ms voltage clamp experiment is shown in Fig 1J (voltage clamp protocol inset), demonstrating the transient behavior of this current. S5 Fig shows the steady-state activation curve, steady-state inactivation curve, and the time constants used in the model and its comparison to values derived from experimental data [26]. The fitted formulas to fully describe *I*_Na_ are shown in Table F of S1 Text.

#### Leakage channel current

The leakage channel current constitutes all ions crossing the cell membrane that are not accounted for [42], using:

$$\begin{array}{c} I_{L,i}=G_{L,i}\left(V-E_{L,i}\right) \end{array}$$ (13)

where *I*_L,*i*_ is the leakage current for Type *i* hMSCs, *G*_L,*i*_ is the conductance of the leakage channel for Type *i* hMSCs, and *E*_L,*i*_ is the leakage reversal potential for Type *i* hMSC (where *i* = A, B, or C). For each type of hMSC, different leakage conductance and reversal potential values were used to satisfy its resting membrane potential (RMP) at approximately -35 mV [9, 26, 27]. A simulation demonstrating the resting potential lies at -35 mV is shown in S6 Fig The formulas to describe the leakage currents are shown in Table G of S1 Text.

#### Ion channel assumptions

Most patch clamp studies are conducted at room temperature (21°C to 22°C) [26, 27]. The Nernst potential, which is dependent on temperature, was therefore extrapolated to normal body temperature (37°C, or 310 K). For the purposes of this study, it is also assumed the hMSC extracellular and intracellular sodium, potassium, and calcium concentrations are constant both in hMSC-hCM coupling and fusion. Finally, it is assumed that no other ionic current plays a significant role in the electrical activity of hMSCs.

### hMSC-hCM Single-Cell Interactions

#### Modeling hMSC-hCM coupling

The hMSC models were coupled to the ten Tusscher endocardial, midcardial, and epicardial healthy hCM models of the ventricular action potential [20] using established cell-cell coupling equations [22]:

$$\begin{array}{c} \frac{dV_{\text{hCM}}}{dt}=-\frac{1}{C_{m,\text{hCM}}}\left[I_{\text{tot},\text{hCM}}+I_{\text{stim}}+I_{\text{gap},\text{hCM}}\right] \end{array}$$ (14)

$$\begin{array}{c} \frac{dV_{\text{hMSC}}}{dt}=-\frac{1}{C_{m,\text{hMSC}}}\left[I_{\text{tot},\text{hMSC}}+I_{\text{stim}}+I_{\text{gap},\text{hMSC}}\right] \end{array}$$ (15)

such that:

$$\begin{array}{c} I_{\text{gap},\text{hCM}}=f_{\text{hMSC}}G_{\text{gap}}\left(V_{\text{hCM}}-V_{\text{hMSC}}\right) \end{array}$$ (16)

$$\begin{array}{c} I_{\text{gap},\text{hMSC}}=G_{\text{gap}}\left(V_{\text{hMSC}}-V_{\text{hCM}}\right) \end{array}$$ (17)

and:

$$\begin{array}{c} f_{\text{hMSC}}=\frac{\%\text{hMSC}}{100-\%\text{hMSC}} \end{array}$$ (18)

where *I*_gap_ is the gap junction current between hMSCs and hCMs, *G*_gap_ is the gap junction conductance, % hMSC is the percentage of hMSCs in a homogeneously distributed hMSC-hCM population, and *V*_*j*_, *C*_m,*j*_, and *I*_tot,*j*_ are the voltage, capacitance, and total current of cell type *j* (where *j* = hCM or hMSC), respectively. The single-cell coupling simulations were performed in a homogeneously distributed hMSC-hCM population ranging from 0% (control) to 80% hMSCs, with basic cycle lengths of 1000 ms. Simulations were run until steady-state was achieved. The characteristics of interest, action potential duration (APD) and plateau height (*V*_*APD*/2_), were defined as time to 90% repolarization and *V*(*t* = *APD*/2), respectively. All simulations were written in MATLAB (The MathWorks, Natick MA) and numerically integrated using a stiff ordinary differential equation solver (ode15s).

#### Modeling passive hMSC-hCM coupling

To model passive hMSCs and their effects on hCMs, *I*_tot,hMSC_ was set equal to zero in Eq 15.

#### Modeling hMSC-hCM fusion

The hMSC models were fused to the midcardial ten Tusscher healthy hCM electrophysiological models by combining respective *I*_tot_ and *C*_m_ terms, such that:

$$\begin{array}{c} \frac{dV_{\text{fused}}}{dt}=-\frac{1}{C_{m,\text{hCM}}+f_{\text{hMSC}}C_{m,\text{hMSC}}}\left[I_{\text{tot},\text{hCM}}+f_{\text{hMSC}}I_{\text{tot},\text{hMSC}}+I_{\text{stim}}\right] \end{array}$$ (19)

where *V*_fused_ is the voltage of the fused cell. To validate this method of modeling cell fusion, we compared our simulations results to the limiting case of cell coupling where *G*_gap_ → ∞, which should theoretically converge to the same answer. This was confirmed in S7 Fig

#### APD restitution curve protocol

A standard S1–S2 APD restitution protocol [20] was performed at the single-cell level on hCMs with 0% hMSC coupling (control), as well as low (5%), moderate (15%), and high (25%) percentages of coupled hMSCs. Briefly, 10 S1 stimuli were applied at a basic cycle length of 600 ms, followed by a single S2 stimulus at a certain diastolic interval (DI) after the last action potential generated.

### Modeling hMSC-hCM Coupling at the Tissue Level

#### Two dimensional cardiac tissue sheet configuration

For the vulnerable window (VW) analysis, a single layer tissue model was used, composed of hCMs and hMSCs. Specifically, a 5 cm × 5 cm two-dimensional midcardial cardiac tissue sheet was simulated, with randomly inserted hMSCs comprising either 0%, 5%, 15%, or 25% of the total cell population (S8 Fig). Three random configurations (i.e., n = 3) were tested for each percentage of hMSC insertion; the mean and standard deviation of VW are reported. A schematic summarizing the geometry, mesh size, and key node characteristics is shown in S8 Fig. Each node executed either the midcardial hCM model membrane kinetics [20] or the selected hMSC membrane kinetics, such that:

$$\begin{array}{c} \frac{\partial V}{\partial t}=-\frac{I_{\text{tot},k}+I_{\text{stim}}}{C_{m,k}}+D_{k,x}\frac{\partial^{2}V}{\partial x^{2}}+D_{k,y}\frac{\partial^{2}V}{\partial y^{2}} \end{array}$$ (20)

for:

$$\begin{array}{c} D_{k,x}=\frac{1}{\rho_{k,x}S_{k,x}C_{m,k}^{\prime }} \end{array}$$ (21)

where $C_{m,k}^{\prime }$ is the cell capacitance per unit surface area of cell type *k*, *S*_*k*,*x*_ is the surface-to-volume ratio of cell type *k* in the *x* direction, and *ρ*_*k*,*x*_ is the effective cellular resistivity of cell type *k* in the *x* direction (where *k* = hCM or hMSC).

Midcardial hCM electrical activity was simulated with the ten Tusscher midcardial model [20], while hMSC electrical activity was simulated with the models herein. Euclidian geometry was selected, with Δ*x* = Δ*y* = 0.01 cm, and Δ*t* = 0.01 ms. Neumann-type boundary conditions were implemented to solve the partial differential equations. As performed elsewhere [43], anisotropy was modeled as *D*_*k*,*x*_ = 4*D*_*k*,*y*_. Relevant parameters for the development of these simulations can be found in Table A of S1 Text. All tissue simulations were written and executed in Python.

#### Stimulation protocol and vulnerable window assessment

A cross-field stimulation protocol was applied, as described elsewhere [43, 44]. Briefly, after achieving steady-state conditions, two S1 stimuli were applied at the left end of the tissue with a basic cycle length of 1000 ms. An S2 stimulation was applied 300–500 ms after the second S1 stimulus, at 1 ms increments, at the bottom left corner of the tissue (1.25 cm wide × 2.5 cm high). For each tissue model, the VW was defined as the range of S1–S2 intervals that led to at least one spiral wave rotation.

#### APD dispersion analysis

A modified version of an established APD dispersion analysis [45] was used on the 5 cm × 5 cm two-dimensional midcardial cardiac tissue sheets described above. APD was calculated after the second S1 stimulus, with a basic cycle length of 1000 ms. APD dispersion, *ζ*, was defined as:

$$\begin{array}{c} \zeta =\sqrt{\frac{1}{N}\sum_{i=1}^{N}{\left(APD_{i}-\bar{APD}\right)}^{2}} \end{array}$$ (22)

where *i* is an hCM grid point, N is the total number of hCM grid points, and $\bar{APD}$ is the mean APD of hCMs in the tissue. Mean and standard deviation of APD dispersion of three random tissue sheet configurations (n = 3) are reported for each type of hMSC inserted at 5%, 15%, and 25% of the total cell population.

#### Conduction velocity restitution curve protocol

A standard S1–S2 CV restitution protocol [20] was simulated for a 1 cm × 1 cm two-dimensional midcardial cardiac tissue monolayer with properties analogous to the larger 5 cm × 5 cm tissue models described above. Briefly, two S1 stimuli were applied at varying DIs, and resultant CVs were measured after the second S1 stimulus. CV was measured in the *x* direction along each row of tissue nodes; mean and standard deviation of CV across the 100 rows is reported.

### Sensitivity Analysis

To quantify the impact of each hMSC parameter on the hCM APD, an established multivariable regression analysis was performed [46, 47]. In 300 trials for each hMSC model, we randomly varied hMSC maximum conductance parameters and time constant parameters by a log-normally distributed pseudorandom scale factor with a standard deviation of 10%, as described elsewhere [48]. hMSCs were coupled to midcardial hCMs in a 1:1 ratio in this analysis. From the changes in the model APD outputs (**Y**) and parameters (**X**), a linear approximation can be made to find the normalized parameter sensitivity vector (**B**), such that **Y** ≈ **XB**. Therefore, a positive or negative sign of B (i.e., an element of **B**) indicates a positive or negative correlation between the parameter of interest and APD, respectively. Furthermore, the magnitude of B indicates the sensitivity of the APD to the parameter of interest. To better demonstrate the sensitivity of the APD output to each hMSC cell type, **B** was scaled by *σ*_APD_, the standard deviation of the APDs for each set of 300 trials for a respective hMSC cell type.

## Results

In this study, three hMSC electrophysiology models were developed based on published experimental data. These three models were subsequently used to develop insight into hMSC-hCM electrical interactions.

### hMSC Electrophysiology Model

Three novel electrophysiological models were developed for the triad of hMSC families based on empirical data [26]. After successfully modeling each type of current expressed in hMSCs (Fig 1), it was necessary to validate the whole-cell models by simulating *I*_tot,A_, *I*_tot,B_, and *I*_tot,C_. Total current whole-cell voltage-clamp simulations of Types A, B, and C hMSCs are shown in Fig 3, along with schematics of functional currents for each cell type [26]. Like experimental recordings [26], our simulation had a conditioning potential of -80 mV, followed by 10 mV voltage steps for 300 ms between -60 mV and 60 mV, and a final holding potential of -30 mV. Overall, fitting individual currents (Fig 1) allowed for ample reconstruction of representative whole cell electrical activity. The simulations for Types A and B hMSCs, both of which possess delayed rectifier-like channel activity, generally agree with the magnitude and behavior of experimental total currents for a wide range of voltage contours [26]. As demonstrated by Li et al., *I*_dr_ at a potential of 60 mV has a standard deviation of approximately 90 pA, and the activation time constant for *I*_dr_ at a holding potential of -80 mV has a substantial standard deviation of approximately 25 ms [26]. Since these deviations affect the amplitude and activation kinetics of Types A and B hMSCs, we performed a sensitivity analysis to determine the impact of these parameters on hCM APD (see Sensitivity Analysis below for details). Type C hMSCs, absent of functional hEAG1 expression, also reproduced the magnitude and form of the experimental voltage-clamp experiments characterizing this hMSC family’s electrophysiological behavior [26]. Therefore, we used each of these hMSC models to predict the direct electrical interactions between hMSCs and hCMs.

### Simulations of hCM-hMSC Coupling and Fusion

The three models developed in this study were each coupled and fused to hCMs to better understand direct cell-cell electrical interactions during hMSC cardiotherapies.

#### Cell coupling and fusion effects on action potential waveform

**Fig 4:**
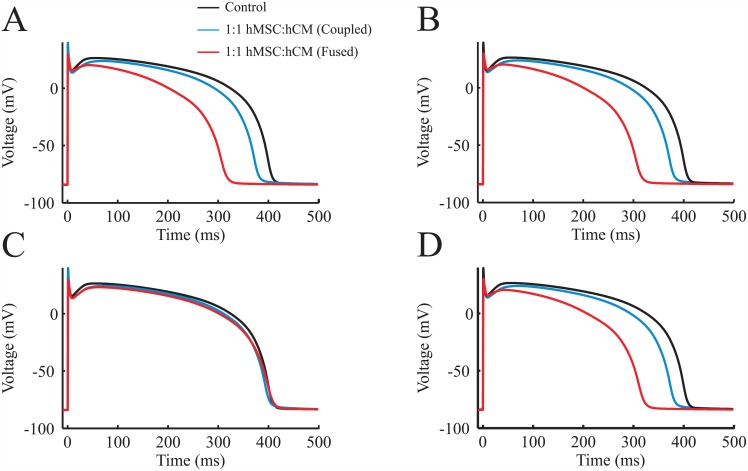
Effects of Direct hMSC-hCM Electrophysiological Interactions on hCM Action Potential. The three hMSC models developed in this study were coupled or fused to midcardial hCM electrophysiological models at a 1:1 cell ratio. (**A**) Type A hMSCs coupled and fused to hCMs resulted in a decrease in hCM APD. (**B**) Type B hMSCs coupled and fused to hCMs resulted in a similar effect. (**C**) Type C hMSCs, absent of delayed rectifier-like hEAG1 channel activity, had a noticeably smaller effect on hCM action potential. (**D**) A mixed population of hMSCs (i.e., Types A, B, and C hMSCs weighted based on the approximate prevalence of 63%, 29%, and 8% in vitro, respectively [26]) coupled and fused to hCMs resulted in a similar effect as Types A and B hMSCs, because only a small portion of hMSCs are Type C [26].

Fig 4 shows the qualitative effects of coupling and fusing midcardial myocytes to each type of hMSC model in 1:1 hMSC-hCM populations (i.e., 50% hMSCs). Types A and B hMSCs expressing functional hEAG1 noticeably shortened the plateau phase, initiated repolarization sooner, and thus distinctively decreased APD when coupling 1:1 with hCMs (Fig 4A and 4B, respectively). Fusing hCMs with Types A and B hMSCs exacerbated APD shortening and lowered the phase 2 plateau voltage compared to the hCM-only control, demonstrating the capability for these mesenchymal cell types to act as major electrical sinks. These effects were evident predominantly during phase 3 of the cardiac action potential, where *I*_K1_, *I*_Kr_, and *I*_Ks_ are most influential (S9 Fig). By contrast, Type C hMSCs absent of delayed rectifier-like hEAG1 currents caused minimal disturbance of the hCM action potential, whether coupled or fused at the 1:1 cell ratio (Fig 4C). To develop an empirically-relevant representation of the impact of hMSCs on APD, a mixed population of hMSCs based on their approximate prevalence [26] was coupled 1:1 to hCMs. As shown in Fig 4D, the mixed population of hMSCs demonstrated similar effects as Types A and B hMSCs. This suggests the relatively benign effects of Type C hMSCs on hCM electrophysiology may be obscured in practice by the other more prevalent hMSC cell types, as only about 8% of hMSCs were characterized as Type C in vitro [26]. Similar trends were evident for coupling between hMSCs and either endocardial or epicardial myocytes, where Type C hMSCs resulted in the least deviation in action potential waveform from the hCM-only control condition (S10 and S11 Figs, respectively).

**Fig 5:**
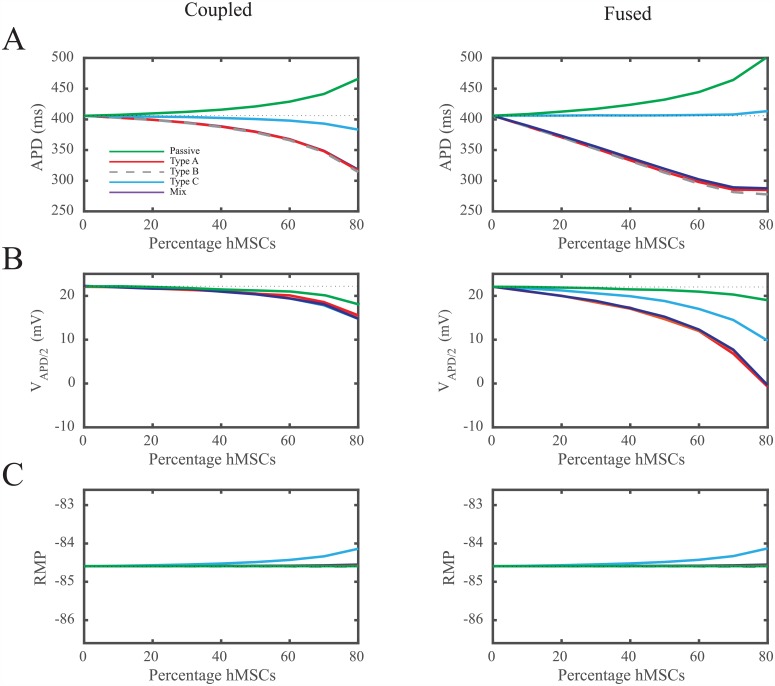
Quantification of hCM Action Potential Waveform Following Direct hMSC-hCM Electrical Interactions. To further explore the effects of each family of hMSC, we quantified the relationship between (**A**) APD, (**B**) *V*_*APD*/2_, (**C**) RMP, and the percentage of coupled (left panel) or fused (right panel) hMSCs in a homogeneously distributed hMSC-hCM population. hMSCs were coupled or fused to midcardial hCMs in an hMSC-hCM population ranging from 0% (control) to 80% hMSCs at 10% increments. These effects were compared to hCMs coupled to a passive R-C circuit-like hMSC cell. In general, Type C hMSCs resulted in the least variation in hCM APD and *V*_*APD*/2_. The dotted lines represent the control condition of hCMs with no hMSC coupling or fusion.

To more clearly demonstrate how the type of hMSC dictates effects on hCM action potential waveform, we quantified the effects of different percentages of hMSCs in a homogeneously distributed hMSC-hCM population in terms of APD, *V*_*APD*/2_, and RMP (Fig 5). As previously observed, coupling hCMs with Type A and B hMSCs with functional delayed rectifier-like hEAG1 channels results in larger deviations in APD compared to Type C hMSCs (Fig 5A, left). Coupling Types A and B hMSCs to hCMs 1:1 (i.e., hMSCs are 50% of the total cell population) decreases midcardial APD by greater than 26 ms, compared to less than 6 ms for Type C hMSCs absent of delayed rectifier-like hEAG1 activity (Fig 5A, left). The effects of Types A and B hMSCs on single hCM APD were exacerbated by cellular fusion (Fig 5A, right). These trends were also evident with action potential plateau height (Fig 5B). By contrast, RMP changed by less than 1 mV in both cellular coupling and fusion conditions (Fig 5C), independent of the hMSC type. In general, it is evident that Type C hMSCs counterbalance the potential passive and electrical sinking natures of hMSCs. This unique cell type consistently results in the least deviation from the action potential of control hCMs. These observations motivated further exploration of why Type C hMSCs act differently, and the interplay between passivity and electrical sinking.

**Fig 6:**
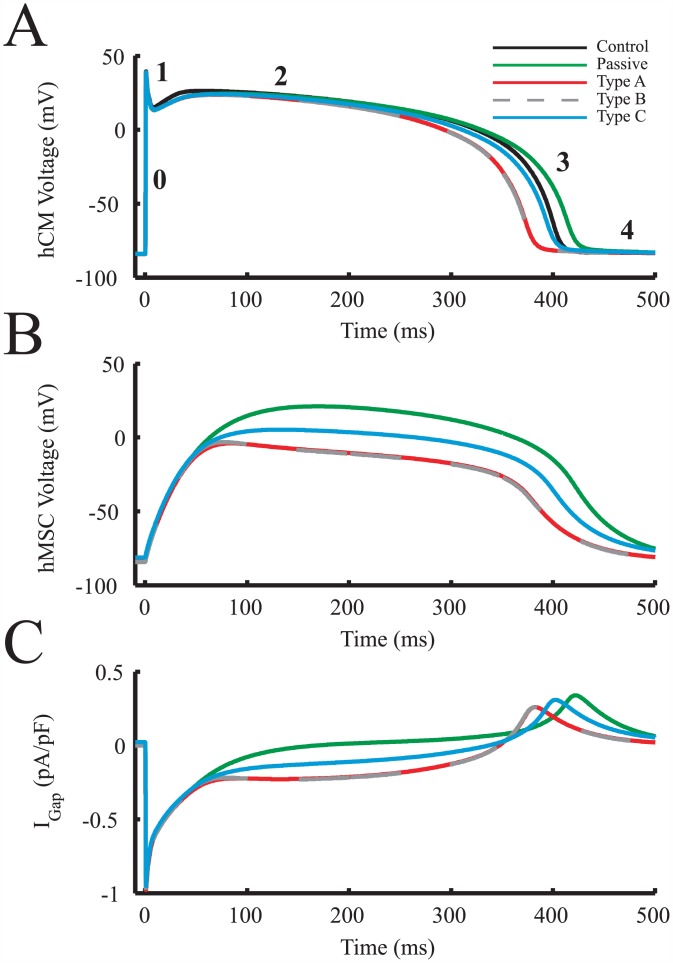
hMSCs Act as Electrical Sources and Sinks in hCM APD Variations. hMSCs were coupled to midcardial hCMs in a 1:1 ratio to examine how hMSC membrane potential and gap current affected the hCM action potential. (**A**) Effects of coupling each type of hMSC on hCM action potentials, compared to hCM-only control. Phases 1 through 4 of the cardiac action potential are labeled for reference. (**B**) hMSC transmembrane voltage throughout an hCM action potential. (**C**) The resulting gap currents betwen hMSCs and hCMs due to differences in voltage between cell types. I_Gap_ was defined as current flowing from the hMSC to the hCM (i.e., I_Gap,hCM_).

hMSCs were coupled to midcardial hCMs in a 1:1 ratio to further study the overall electrical source and sink behavior of each type of hMSC and its role in altering hCM action potentials (Fig 6A). In this configuration, we examined the dependence of hMSC membrane potential and gap current (Fig 6B and 6C, respectively) on the hMSC type, which ultimately influences the hCM action potential. As shown in Fig 6B, the membrane potential of types A and B hMSCs were lower than both type C and passive hMSCs throughout an action potential. As a result, they demonstrated a larger sinking gap current than hMSCs absent of delayed rectifier current activity throughout phase 2 of the action potential, leading to shortened hCM APDs. Type C hMSCs had a membrane potential that was intermediate between passive and delayed rectifier-acting hMSCs (Fig 6B), corresponding to a favorable gap current (Fig 6C) and minimal effects on the coupled hCM APD. In general, lower hMSC membrane potentials led to larger sinking effects and therefore shorter APDs. To identify the culprits of lower hMSC membrane potentials and thus larger sinking effects, we further examined outward currents of each hMSC cell type.

**Fig 7:**
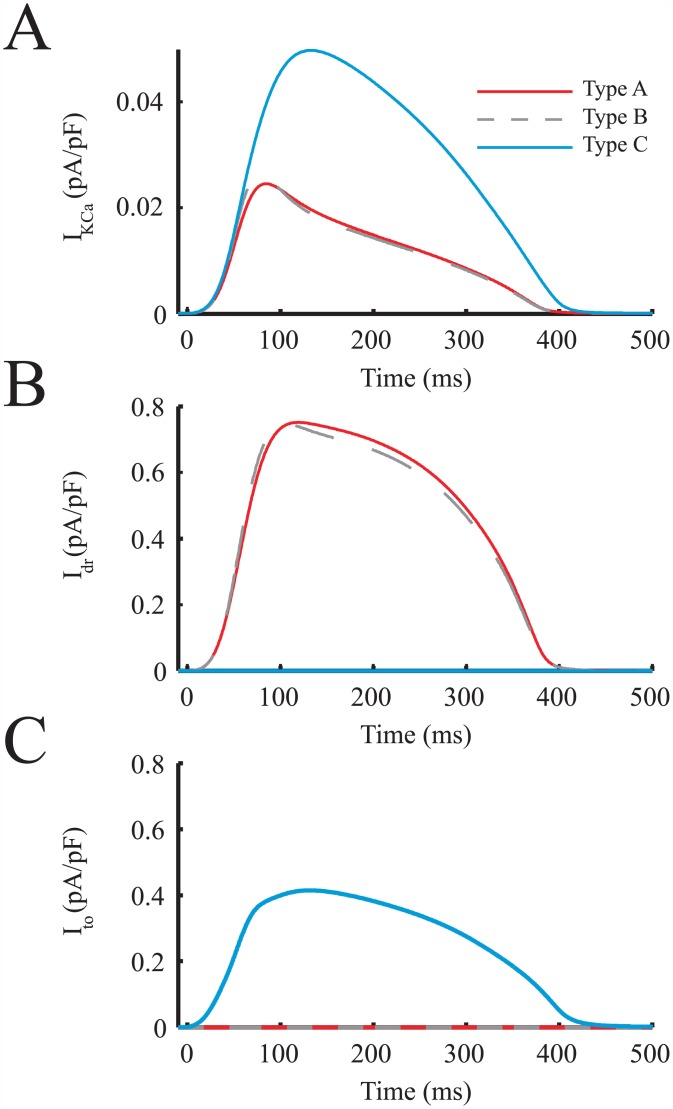
Key hMSC Outward Currents Involved in Electrical Sinking. The main hMSC outward currents involved in a faster initiation of phases 3 and 4 of hCM action potentials were examined. The hMSC currents analyzed were: (**A**) I_KCa_, (**B**) I_dr_, and (**C**) I_to_. During an hCM action potential, I_dr_ had the largest magnitude and area under the curve, which resulted in the greatest electrical sink effects.

The main hMSC outward currents potentially triggering an earlier initiation of phases 3 and 4 of hCM action potentials were examined in the same 1:1 hMSC:hCM ratio (Fig 7). Specifically, I_KCa_, I_dr_, and I_to_ were examined (Fig 7A, 7B and 7C, respectively). During an hCM action potential, the delayed-rectifier like current of Types A and B hMSCs had the highest outward current magnitude and area under the curve; this resulted in the largest electrical sinking effects by restraining the depolarization of hMSCs in synchrony with hCMs. The peak hEAG1 current (I_dr_) was two-fold greater than the maximum magnitude of I_to_, and nearly twenty-fold greater than the maximum magnitude of I_KCa_. The greater net outward current of Types A and B hMSCs resists these hMSCs from approaching the transmembrane voltage of hCMs, thus resulting in an overall larger sinking effect. To confirm the substantial role of hEAG1 outward currents in shortening APD, a quantitative sensitivity analysis was performed.

#### Sensitivity analysis

**Fig 8:**
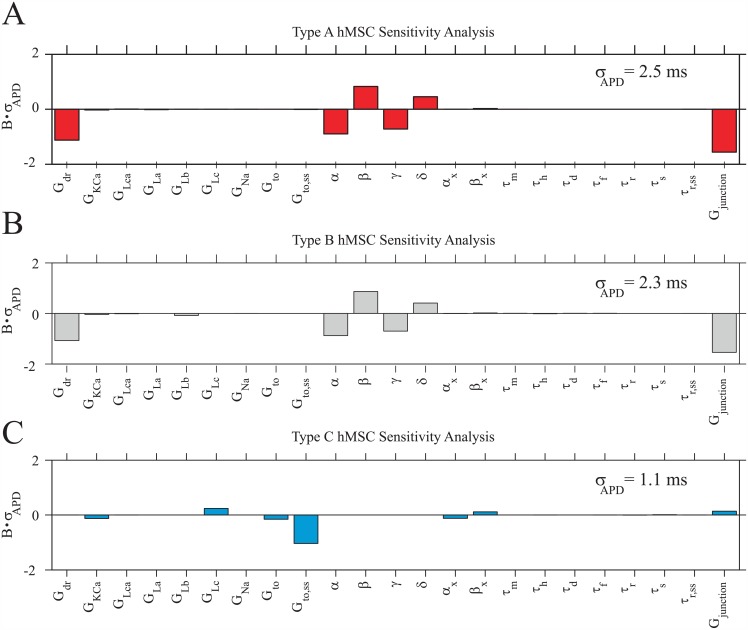
hCM APD Sensitivity to hMSC Current Parameters. A sensitivity analysis based on 300 trials per cell type was performed to develop insight into the correlation between hCM APD and current parameters of (**A**) Type A hMSCs, (**B**) Type B hMSCs, and (**C**) Type C hMSCs, each at a 1:1 ratio with midcardial hCMs. hCM APD was most sensitive to Types A and B hMSC gap junctional conductance (G_junction_) and delayed-rectifier current parameters (*G*_*dr*_, *α*, *β*, *γ*, and *δ*). hCM APD was less sensitive to Type C hMSC G_junction_, demonstrating that this cell type’s effects are less disruptive to the APD of coupled hCMs and caused less APD variability (i.e., lowest value of *σ*_APD_, as noted in each panel). The normalized parameter sensitivity vector, **B**, was scaled by *σ*_APD_ to better illustrate the sensitivity of the APD output to each hMSC cell type.

A sensitivity analysis was performed for Types A, B, and C hMSCs in 1:1 ratios with midcardial hCMs to confirm the role of hEAG1 currents, and to develop insight into how sensitive hCM action potentials are to direct hMSC-hCM coupling (Fig 8). Coefficients of determination of 0.99, 0.99, and 0.98 (for Types A, B, and C hMSCs, respectively) demonstrate the accuracy of using these linear approximations to develop a relationship between input parameters and output APD. hCM APD was negatively correlated and highly sensitive to Types A and B hMSC G_junction_, verifying the large sinking effects of these cell types (Fig 8A and 8B). This sensitivity was amplified by a negative correlation with G_dr_ and its activating parameters (*α* and *γ*), and a positive correlation with its inactivating parameters (*β* and *δ*), demonstrating the influential role of hEAG1 currents on APD. The variations of these parameters were almost exclusively responsible for the more than two-fold greater *σ*_APD_ compared to Type C hMSCs (*σ*_APD_ values indicated in Fig 8). Therefore, hEAG1 activity is indeed the leading cause of sinking effects of hMSCs, and results in larger deviations in APD versus Type C hMSCs.

This sensitivity analysis also gives insight into how well controlled the process of direct hMSC-hCM coupling can be in affecting hCM APD. The larger *σ*_APD_ for both Types A and B hMSC sensitivity analyses suggest that transplanted cells with delayed rectifier-like currents may be less consistent in their effects on hCM APD. Furthermore, as previously described, the activation kinetics of hEAG1 currents are highly variable [26]. Therefore, this large variability, coupled with the high sensitivity of APD to these channel parameters, could be detrimental in controlling hMSC electrical effects in a cardiac implant setting. hCM APD is also sensitive to Types A and B G_junction_, which is itself intrinsically variable [15], further contributing to poor electrical stability. Overall, Type C hMSCs appear to induce the least variations in hCM APD.

### Vulnerable Window Analysis

**Fig 9:**
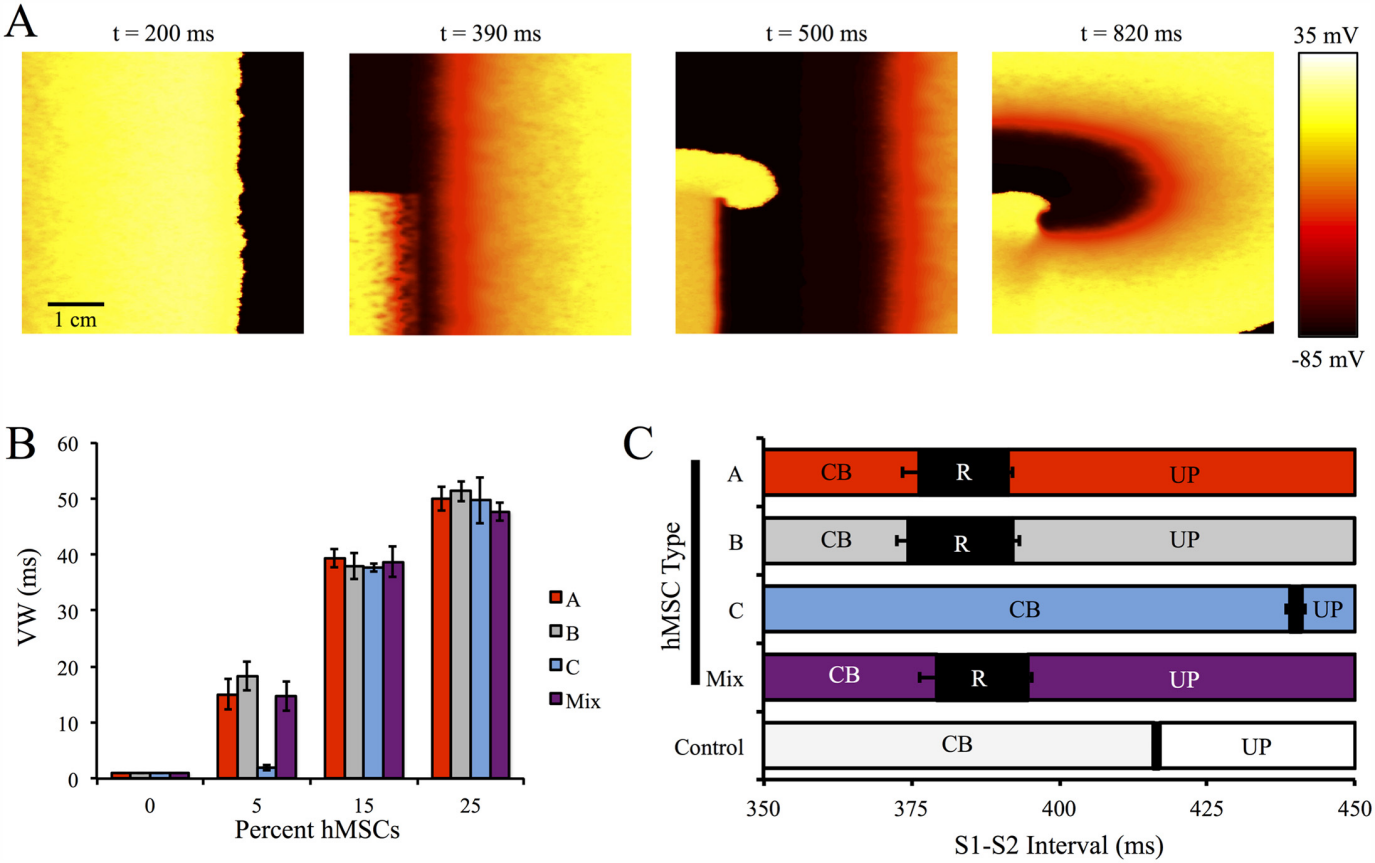
VW Analysis on Cardiac Tissue With Randomly Inserted hMSCs. A VW analysis was performed to better understand the pro-arrhythmic potential of hMSC insertion in cardiac tissue. (**A**) Selected frames from a representative simulation of an S1–S2 interval of 380 ms that led to re-entry with 25% Type A hMSC random insertion into 2-D cardiac tissue. (**B**) The VWs for tissues with 0% (control), 5%, 15%, and 25% random insertion of hMSCs; at low levels of hMSC insertion, Type C hMSCs lead to the smallest increase in VW compared to control. (**C**) Analysis of S1–S2 intervals leading to conduction block (CB), re-entry (R), or uninterrupted propagation (UP) with 5% hMSC insertion. Error bars represent standard deviation based on three tissue configurations per condition.

To understand the arrhythmogenic effects of direct hMSC-hCM coupling at the tissue level, a VW analysis was performed on a single layer, anisotropic 5 cm × 5 cm 2-D midcardial tissue with 0% hMSCs (healthy control), 5%, 15%, or 25% randomly inserted hMSCs repeated for three different configurations per condition (see Fig 9A and S1–S4 Videos for sample re-entry simulations at selected S1–S2 intervals).

As shown in Fig 9B, VWs lengthened with increasing percent of inserted hMSCs. Interestingly, at low (5%) insertion levels, VWs were dependent on the type of coupled hMSCs (Fig 9B); inserting hMSCs with delayed rectifier-like activity (i.e. Types A, B, and mixed populations of hMSCs) led to substantially larger VWs (approximately 15 to 20 ms) compared to Type C hMSCs (VW = 2.0 ± 0.5 ms, n = 3). At greater levels of hMSC insertion (i.e., 15% and 25%), VWs were nearly independent of the type of coupled hMSCs, and VWs exceeding 50 ms were observed. The S1–S2 intervals that led to re-entry for each hMSC type at low levels of insertion are shown in Fig 9C. As expected, the shifts in S1–S2 intervals leading to re-entry depended on the different mean tissue APDs (S12 Fig).

### Dispersion of Refractoriness and Restitution Analysis

**Fig 10:**
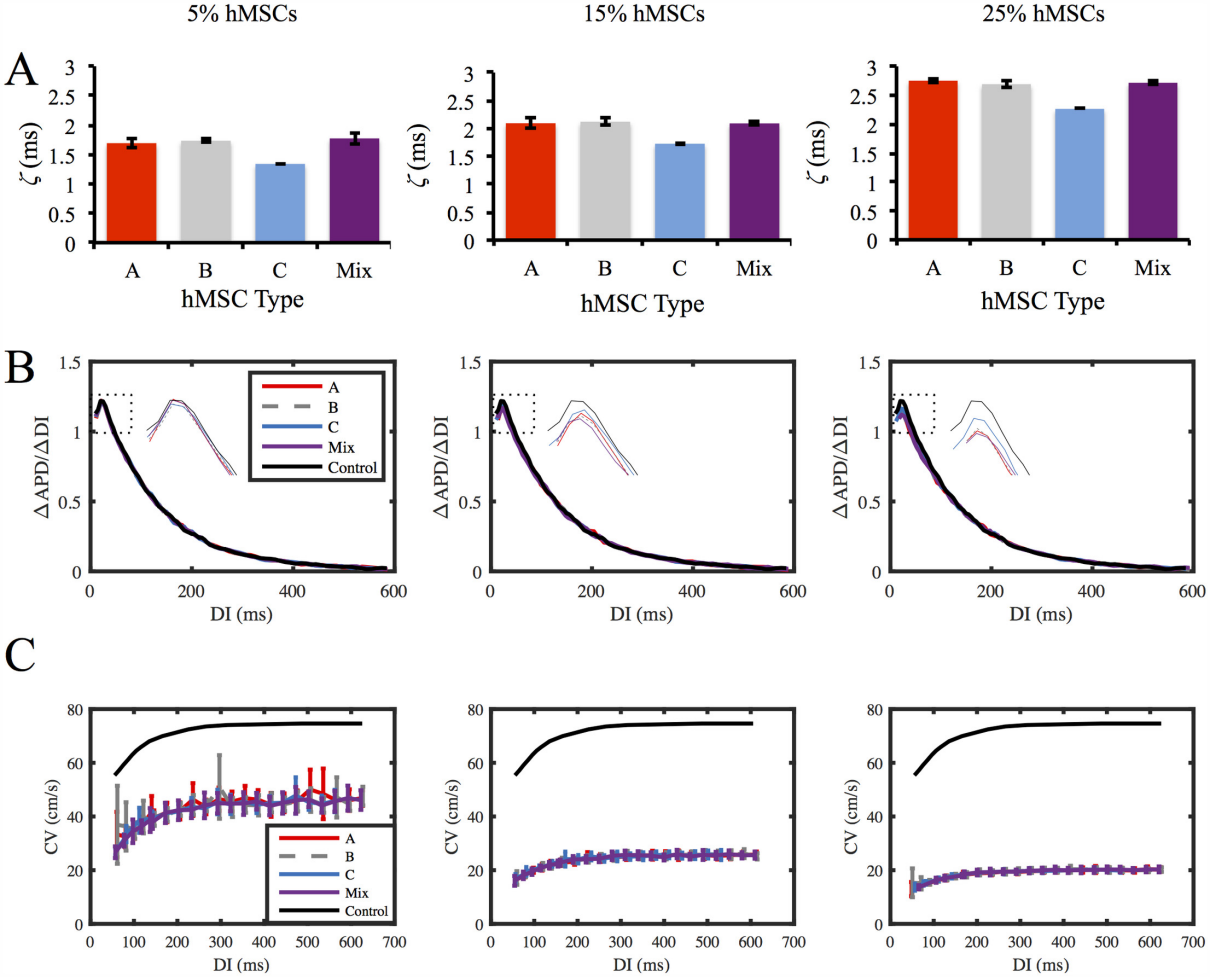
hMSC Effects on Dispersion of Refractoriness and Restitution Curves. Effects of the types of hMSCs coupled, as well as the percentage of hMSCs coupled, on dispersion of refractoriness and restitution curves were examined. (**A**) APD dispersion (*ζ*) was lowest for Type C hMSCs. APD dispersion increased with higher percentages of coupled hMSCs. (**B**) APD restitution slopes, as well as the range of DIs for slopes greater than 1, decreased with hMSC percentage. Inset plot shows expanded region of APD restitution slopes greater than 1. (**C**) CV restitution curves were independent of hMSC type. As the percentage of inserted hMSCs increased, the maximum CV decreased.

Various modeling studies have demonstrated APD dispersion may influence re-entry [45, 49], while APD restitution slope, the range of DIs for APD restitution slopes greater than 1, and CV restitution slope are key factors in restitution-induced instability [20, 50–53]. Fig 10 illustrates the effects of the percentage and types of hMSCs on each of these arrhythmogenic factors.

As expected, APD dispersion (*ζ*) increased with greater levels of hMSC insertion for all hMSC types (Fig 10A). However, APD dispersion was approximately 21%, 18%, and 17% lower for Type C hMSCs compared to hMSCs with delayed rectifier-like activity at 5%, 15%, and 25% hMSC insertion, respectively (Fig 10A). S12 Fig shows APD maps for cardiac tissues with 5% hMSC insertion.

APD restitution slopes, as well as the range of DIs for slopes greater than 1, were slightly decreased following coupling with each hMSC type (Fig 10B; for raw APD restitution curves, see S13 Fig). Even at 25% hMSC insertion, the shift in maximum APD restitution slope was less than 10% (Fig 10B). CV restitution slopes markedly decreased following hMSC insertion, by as much as 71% at 25% hMSC insertion (Fig 10C). The coupling effects on CV restitution slopes were predominately dependent on percentage of hMSC inserted, rather than the type of hMSC (Fig 10C). Altogether, this dispersion of refractoriness and restitution analysis supports the claim that increased arrhythmogenic potential of inserted stem cells is minimized by Type C hMSCs at low levels of hMSC insertion, as VW and APD dispersion are lowest for this cell type, without adversely affecting APD and CV restitution slopes in comparison to delayed rectifier-like hMSCs.

## Discussion

Our study provides insight into hMSC electrical activity, and the electrophysiological effects when directly coupling hMSCs to hCMs at both the single-cell and the tissue level. This computational analysis allowed us to hypothesize an electrophysiology-based approach for improved hMSC-based cardiotherapies, which has not been suggested elsewhere. We first developed three novel hMSC electrophysiology models based on a published empirical triad of hMSC families having distinct ion channel currents: Type A) I_dr_ and I_KCa_; Type B) I_dr_, I_KCa_, I_Na_, and I_LCa_; and Type C) I_KCa_ and I_to_ [26, 28]. Subsequently, each hMSC model type was coupled to an adult ventricular hCM electrophysiology model to better understand the direct interactions of these cell types. The computational analysis led us to find that: 1) our model simulations are consistent with a range of empirical findings; 2) hMSC-hCM direct coupling can increase vulnerability to re-entry; and 3) vulnerability to re-entry can be minimized using Type C hMSCs at low levels of stem cell insertion.

### Comparison of Model Results to Empirical Findings

The ability of our computational models to reproduce empirical electrical hMSC and hMSC-hCM co-culture findings supports the validity of our results. As previously described, fitting individual currents (Fig 1) allowed reconstruction of representative whole cell voltage-clamp data by Li et al. (Fig 3) [26]. This enabled us to simulate hMSC-hCM coupling to develop insight into direct electrical effects of co-culturing these two cell types.

The complex hMSC-hCM interactome, which also includes paracrine signaling [6, 11], makes it empirically infeasible to isolate direct electrophysiological coupling effects on APD. For example, Askar et al. have previously shown that the hMSC secretome alone significantly increases neonatal rat cardiomyocyte APD and significantly decreases Cav1.2 and Kv4.3 levels [54], while DeSantiago et al. demonstrated the hMSC paracrine factors stimulate the L-type calcium channel current and calcium transient activity in mouse ventricular myocytes [55]. Furthermore, Askar et al. found the paracrine effects on APD to be dose-dependent [54]. Several studies [13, 16, 54] demonstrate that hMSC co-culture does not lead to APD shortening in vitro, whereas our model studies suggest direct hMSC-hCM coupling alone would tend to shorten APD. Therefore, we hypothesize that in the experimental setting, hMSC-mediated paracrine effects may overshadow the model-predicted APD shortening effects of direct heterocellular coupling.

Furthermore, the hMSC secretome reportedly alters atrial myocyte conduction [56], but does not significantly affect the conduction of ventricular myocytes [54], making it reasonable to compare our model results to empirical conduction and VW findings. Studies have shown that sufficient hMSC supplementation decreases CV and CV restitution slopes [16, 54], consistent with our simulations. Specifically, Chang et al. observed co-culturing cardiomyocytes with greater than 10 percent of hMSCs decreased CV and the CV restitution slope [16]. Moreover, sufficient hMSC supplementation increased inducibility of re-entry [16], which was also shown in our simulations (Fig 9). Based on our direct coupling-only simulations reproducing empirical co-culture conduction and VW findings, we hypothesize that in the experimental setting, hMSC-mediated paracrine effects on hCM conduction do not counteract the effects of direct heterocellular coupling demonstrated in this study, emphasizing the importance of understanding and minimizing the potential sources of hMSC-related arrhythmogenicity.

### Mechanistic Insights into hMSC-hCM Coupling

Despite their non-excitable nature, hMSCs express gap junction proteins [15] and are therefore capable of influencing hCM action potentials. Furthermore, these effects cannot be presumed to be simply passive, as shown in Figs 5 and 6A. In the case of a passive cell, there is a consistent increase in hCM APD. The relatively large capacitance of hMSCs (approximately 60 pF [26], compared to 6.3 pF for cardiac fibroblasts [22]) makes this effect substantial, resulting in increases in APD of approximately 50 ms with a population of 80% passive hMSCs with midcardial hCMs.

These passive effects were not duplicated once the cells expressed their respective ionic currents. Unlike passive hMSCs, Types A and B hMSCs decreased APD independent of hCM cell type. For example, the APDs of midcardial hCMs shortened by approximately 88 ms with a population of 80% Type A or B hMSCs. This effect was exacerbated in cellular fusion, where midcardial hCM APD was shortened by approximately 120 ms. During an hCM action potential, the peak hEAG1 current was two-fold greater than the maximum magnitude of I_to_, and nearly twenty-fold greater than the maximum magnitude of I_KCa_. The larger outward current of Types A and B hMSCs resists hMSCs from approaching the transmembrane voltage of hCMs, resulting in an overall larger sinking effect that shortens phase 2 of the cardiac action potential, and initiates phases 3 and 4 of repolarization earlier. Such drastic changes in the action potential waveform could be possible in vivo if the delivered stem cells cluster in regions of the heart [57], such that hMSCs outnumber hCMs locally. This would be of even greater concern if the high incidence of cell fusion reported in vitro [10] were also found to occur in vivo as suggested by recent animal studies [58]. The implications of action potential variations include pathological electrical and mechanical states.

### Potential Pathophysiological Consequences of hMSC-hCM Coupling

Overall, our simulations suggest hMSC-hCM coupling: 1) alters action potential waveform at the single-cell and tissue level; 2) increases dispersion of APDs at the tissue levels; and 3) substantially decreases CV. Shortening of APDs by Types A and B hMSCs could have notable electrophysiological implications in the heart. Studies have shown that shortening of APDs could induce ventricular tachycardias, suggesting Types A and B hMSCs may be capable of pro-arrhythmic electrical remodeling [17, 59, 60]. Furthermore, one signature of ischemic patients is a loss of epicardial action potential dome, resulting in ST-segment elevation [61]. hMSC direct coupling to hCMs could exaggerate these effects by clustering in the epicardium and acting as an electrical sink, thus becoming pro-arrhythmic.

Substantial decreases in APD due to Types A and B hMSCs could also portend altered Ca^+2^ transients in the hCM, resulting in decreased inotropy [62–67]. Such alterations could directly impact left-ventricular pressure development [22], which is of particular concern for myocardial infarction patients who already suffer decreases in ejection fraction, preload, stroke work, rate of pressure development, and overall mechanical efficiency [68].

The large variability in electrical activity of Types A and B hMSCs presents another potential source of arrhythmogenicity. hCM APD was negatively correlated and highly sensitive to Types A and B hMSC G_junction_ (Fig 8A and 8B). This gap conductance has been shown empirically to be highly variable with a coefficient of variation of 87% [15]. The potentially irregular actions of Types A and B hMSCs are further amplified by the fact that hEAG1 activation kinetics are also highly variable, with a coefficient of variation of approximately 35% [26]. Since there is a highly negative correlation between hCM APD and numerous I_dr_ components (e.g., its activating parameters and G_dr_), and a highly positive correlation with its inactivating parameters, hMSCs with delayed rectifier-like currents are likely to be unpredictable in their direct effects on hCM APD. This is exacerbated by the fact that hMSC insertion leads to increased APD dispersion in a dose-dependent manner, which could unfavorably alter VWs and electrical stability [45, 49].

Decreased CV caused by hMSC supplementation (Fig 10C) is an established source of re-entrant loops [16], making hMSC-hCM direct coupling potentially arrhythmogenic. Chang et al. showed the potential of re-entrant arrhythmias in vitro was dependent on hMSC supplementation [16], which was confirmed in our simulations (Fig 9B). The decrease in CV is more drastic with increased hMSC supplementation (Fig 10C), which may occur if hMSCs cluster in a localized region, resulting in an increased probability for re-entry. Decreased CV also plays a significant role in ischemic patients. Specifically, ischemic patients also have signatures of transmural conduction slowing, resulting in ST-segment elevation and T-wave inversion [61]. These abnormalities may be exacerbated by the decreased CV effects of hMSC insertion.

### Implications of Findings for Future Cardiotherapies

Current hMSC cardiotherapies involve implementation of electrically-unspecified hMSCs. As a result, Types A and B hMSCs, which reportedly account for a majority of hMSCs [26], will tend to dominate the electrical interactions with hCMs. This was seen in Figs 4 and 5, where the mixed population of hMSCs acted almost indistinguishably from Types A and B hMSCs. This model study suggests that the isolation of Type C hMSCs, absent of delayed rectifier-like currents, may offer superior effectiveness and safety as a cell-based cardiotherapy at low levels of hMSC insertion by minimizing VWs and action potential waveform perturbations compared to other hMSC types. Type C hMSCs exhibited unique electrical activity that was intermediate between the passive and delayed rectifier-functioning hMSCs, resulting in a favorable gap current. The equilibrating source-sink actions within the Type C hMSC gap currents resulted in smaller deviations in the APD (Figs 4 and 5), corresponding to longer action potential wavelengths at the tissue level following hMSC insertion (Table H of S1 Text), which we hypothesize contributed to this cell type having the smallest VW at low levels of hMSC insertion (Fig 9B and 9C). This suggests a decreased likelihood of the potential adverse electrical effects previously described.

It is also important to note that overall at the tissue level, the VW increased at greater levels of hMSC-hCM direct coupling, and became independent of hMSC type at moderate and high levels of hMSC insertion. Previous findings suggest the hMSC-hCM interactome involves not only intrinsic, direct cell-cell coupling, but also indirect paracrine signaling through the release of largely unidentified soluble factors and exosome nanovesicles [6, 11]. Harnessing and delivering the key components of the hMSC secretome while circumventing the potentially pro-arrhythmic effects of direct cell-cell coupling may offer a superior cardiac therapy in the future.

### Limitations and Future Work

We note several limitations of the hMSC models developed. As previously discussed, the activation time constant for *I*_dr_ at a holding potential of -80 mV has a coefficient of variation of approximately 35% [26]. This variability affects the output APD, as suggested by the sensitivity analysis, demonstrating the necessity for further empirical investigation into the kinetics of hMSC *I*_dr_. We also assumed that only a triad of families of hMSCs exist, but there may be more; for instance, it has been reported that ion channel expression varies with cell cycle progression [69–71], which may contribute to the variable electrical families and activities of hMSCs. However, the limited understanding of this behavior in the context of hMSCs motivated us to focus only on the three previously characterized hMSC families. We also assume constant ionic concentrations across the hMSC cell membrane. Currently, there is not enough experimental data to sufficiently model intracellular calcium levels in hMSCs. Our sensitivity analysis demonstrates that APD is not highly influenced by channels that are largely affected by these variations (e.g., *I*_KCa_), justifying this assumption. Collecting more electrophysiological data on carrier proteins within hMSCs [29] would encourage incorporating transient behavior of ionic concentrations into our models.

A second limitation was that we assumed healthy hCMs in order to develop insight into the arrhythmogenic effects of hMSC insertion into healthy cardiac tissue, effectively performing an in silico Phase I clinical trial. However, we did not consider the effects of microfibrosis or random microscale obstacles [24, 72–77]. Each of these effects was purposely not considered in this study, as hMSC paracrine effects are expected to have a major impact on these changes [78–81]. The simulations performed in this study provide a framework for future investigation into each of these factors.

Therapeutic hMSCs can disperse to both healthy and ischemic regions of the heart, motivating investigation of the effects of hMSC coupling with ischemic hCMs. This healthy hCM-only assumption made it appropriate to model local cardiac behavior (i.e., 5 cm × 5 cm heterogeneous anisotropic tissue) rather than whole heart behavior. Studying the effects of various spatial distributions of hMSCs using a fully three-dimensional anatomically detailed model of the heart could represent an area for future investigation building on the electrophysiology models developed herein.

A fourth limitation was that we did not model other factors that may influence electrical instability, such as short-term cardiac memory and intracellular calcium dynamics [20, 82–84]. Instead, we prioritized other established factors of instability (e.g. APD dispersion, APD restitution slopes, CV restitution slopes), and found several advantages of Type C hMSCs compared to the other mesenchymal stem cell families.

Finally, we assumed no interplay between paracrine signaling and electrophysiological coupling. However, it was recently shown that paracrine signaling can cause upregulation of Cx43 and increase intercellular conduction in atrial myocytes [56], as well as alter ion channel activity in ventricular myocytes [54]. We neglected paracrine mechanisms in our models, so investigating this time-dependent interaction would require further study.

Based on these limitations, areas for future work include: 1) improving the models based on advancements in empirical data on hMSC electrophysiology; 2) considering the effects of microfibrosis or random microscale obstacles in combination with hMSC anti-fibrotic paracrine effects; 3) examining the electrical and electromechanical effects of hMSC models coupled with ischemic hCM models [85]; 4) modeling the interplay between electrophysiological effects and paracrine signaling in the hMSC-hCM interactome; and 5) empirically confirming our simulations, demonstrating that Type C hMSCs minimize the impact on APD and reduce the VW at low levels of hMSC insertion, offering a potential strategy for improving the safety of cardiac cell therapies.

In conclusion, our study provides novel electrophysiological models of hMSCs that reproduce key experimental measurements from patch clamp studies, identifies mechanisms underlying the arrhythmogenic effects of hMSCs coupled to hCMs via gap junctions, underscores the electrical effects associated with hMSC-hCM fusion, and establishes the possibility of isolating a specific sub-population of hMSCs absent of hEAG1 delayed rectifier-like channel activity for minimizing the arrhythmogenic risk of future hMSC-based cell delivery cardiotherapies using low levels of hMSC coupling.

## Supporting Information

S1 Text Supplementary Methods and Tables. Details on numerical methods used for data fitting and solving differential equations can be found. Tables of channel current model formulas are also accessible here.
(DOCX)

S1 Fig I_KCa_ Steady-State Activation and Time Constant Curves. (DOCX)

S2 Fig I_dr_ Data Fitting Curves. (DOCX)

S3 Fig I_LCa_ Steady-State Functions and Time Constant Curves. (DOCX)

S4 Fig I_to_ Steady-State Functions and Time Constant Curves. (DOCX)

S5 Fig I_Na_ Steady-State Functions and Time Constant Curves. (DOCX)

S6 Fig Confirming the Resting Membrane Potential of Each hMSC Model. (DOCX)

S7 Fig Confirming the hMSC-hCM Fusion Model. (DOCX)

S8 Fig Configuration of the Heterogeneous Anistropic hMSC-hCM 2-D Tissue. (DOCX)

S9 Fig Underlying Effects of hMSCs on hCM Ionic Currents During an Action Potential. (DOCX)

S10 Fig Effects of hMSC Coupling and Fusion on Endocardial hCMs. (DOCX)

S11 Fig Effects of hMSC Coupling and Fusion on Epicardial hCMs. (DOCX)

S12 Fig APD Map of Cardiac Tissue With 5% hMSCs Inserted. (DOCX)

S13 Fig Raw APD Restitution Curves Following 5%, 15%, and 25% hMSC-hCM Coupling. (DOCX)

S1 Video Re-Entry With 25% Type A hMSCs Randomly Inserted in Cardiac Tissue. (MP4)

S2 Video Re-Entry With 25% Type B hMSCs Randomly Inserted in Cardiac Tissue. (MP4)

S3 Video Re-Entry With 25% Type C hMSCs Randomly Inserted in Cardiac Tissue. (MP4)

S4 Video Re-Entry With 25% Type D hMSCs Randomly Inserted in Cardiac Tissue. (MP4)
