# Supplementary material for: Modeling Electrophysiological Coupling and Fusion between Human Mesenchymal Stem Cells and Cardiomyocytes
Source: PLoS Comput Biol. 2016 Jul 25;12(7):e1005014. doi: 10.1371/journal.pcbi.1005014 (PMC4959759; doi:10.1371/journal.pcbi.1005014)
Supplement: S1 Text — Details on numerical methods used for data fitting and solving differential equations can be found. Tables of channel current model formulas are also accessible here. (DOCX) [file pcbi.1005014.s001.docx]

# S1 Text: Supplementary Methods and Tables:

***Supplementary Methods:***

*Handling Experimental Data for hMSC Model Development*

Published experimental hMSC electrophysiology data from Li et al. [1] was digitized to develop the hMSC model. Mean I-V I_KCa_ curve data from Figure 4B of Li et al. [1] was defined as the difference between control and 100 nM Iberiotoxin conditions. Mean I-V I_dr_ curve data from Figure 6B of Li et al. [1] was defined as control values. Mean I-V I_LCa_ curve data from Figure 3C of Li et al. [1] was defined as control values. I-V I_to_ curve data from Figure 5A of Li et al. [1] was defined as the difference between control and 4-AP conditions. Mean I-V I_Na_ curve data from Figure 2C of Li et al. [1] was defined as control values. Mean I_dr_ activation data was taken as control values from Figure 6D of Li et al. [1] Mean I_to_ inactivation data was taken from Figure 5B of Li et al. [1] All other activation and inactivation data were derived from respective I-V plots and voltage-clamp data of Li et al. [1] Activation parameters were derived from voltage-clamp data, and were defined as time to reach 63% of the maximum current. Inactivation parameters were derived from voltage-clamp data, and were defined as time to reach I_steady-state_ + 0.37*(I_max_-I_steady-state_), such that time was greater than time of maximum current.

*Computational Methods for hMSC Model Development*

Due to the form of ionic channel equations, both an ordinary differential equation solver and a nonlinear optimization programming algorithm were necessary to fit the hMSC ion channel data. The “ode15s” and “fmincon” MATLAB functions were used as the ordinary differential equation solver and nonlinear optimization programming algorithm, respectively. The ion channel equations with fitted model parameters are given in Tables B-G of S1 Text.

*Modeling hMSC Cellular Resistivity*

Empirical data was unavailable for the cellular resistivity of an hMSC. Therefore, the cellular resistivity of an hMSC ($\rho_{hMSC}$) was calculated by extending the hCM single-channel gap conductance and cellular resistivity relationship [2, 3] to the hMSC cell single-channel gap conductance [4]. This inverse relationship, as described elsewhere [2, 3], is:

$$\rho_{hMSC}g_{gap}=\frac{\pi r_{c}^{2}}{L_{gap}}$$

where $g_{gap}$ is the single channel conductance, $r_{c}$ is the channel radius, and $L_{gap}$ is the length of the gap junction. The length and radius of the hMSC-hCM and hMSC-hMSC gap junctions were assumed to be the same as the hCM-hCM gap junction.

# Supplementary Text References:

[1] Li GR, Sun H, Deng X, Lau CP. Characterization of ionic currents in human mesenchymal stem cells from bone marrow. Stem cells (Dayton, Ohio). 2005 Mar;23(3):371–382.

Available from: http://www.ncbi.nlm.nih.gov/pubmed/15749932.

[2] Jongsma HJ and Wilders R. Gap junctions in cardiovascular disease. Circulation Research, 86(12):1193–7, Jun 2006.

[3] Jongsma HJ and Wilders R. Limitations of the dual voltage clamp method in assaying conductance and kinetics of gap junction channels. Biophysical Journal, 63(4):942–953, Oct 1992.

[4] Valiunas V, Doronin S, Valiuniene L, Potapova I, Zuckerman J, Walcott B, et al. Human mesenchymal stem cells make cardiac connexins and form functional gap junctions. The journal of physiology. 2004 Mar;555(Pt 3):617–626. Available from: http://www.ncbi.nlm.nih.gov/pubmed/14766937.

***Supplementary Tables:***

**Table A: Parameter Values For Modeling hMSCs**


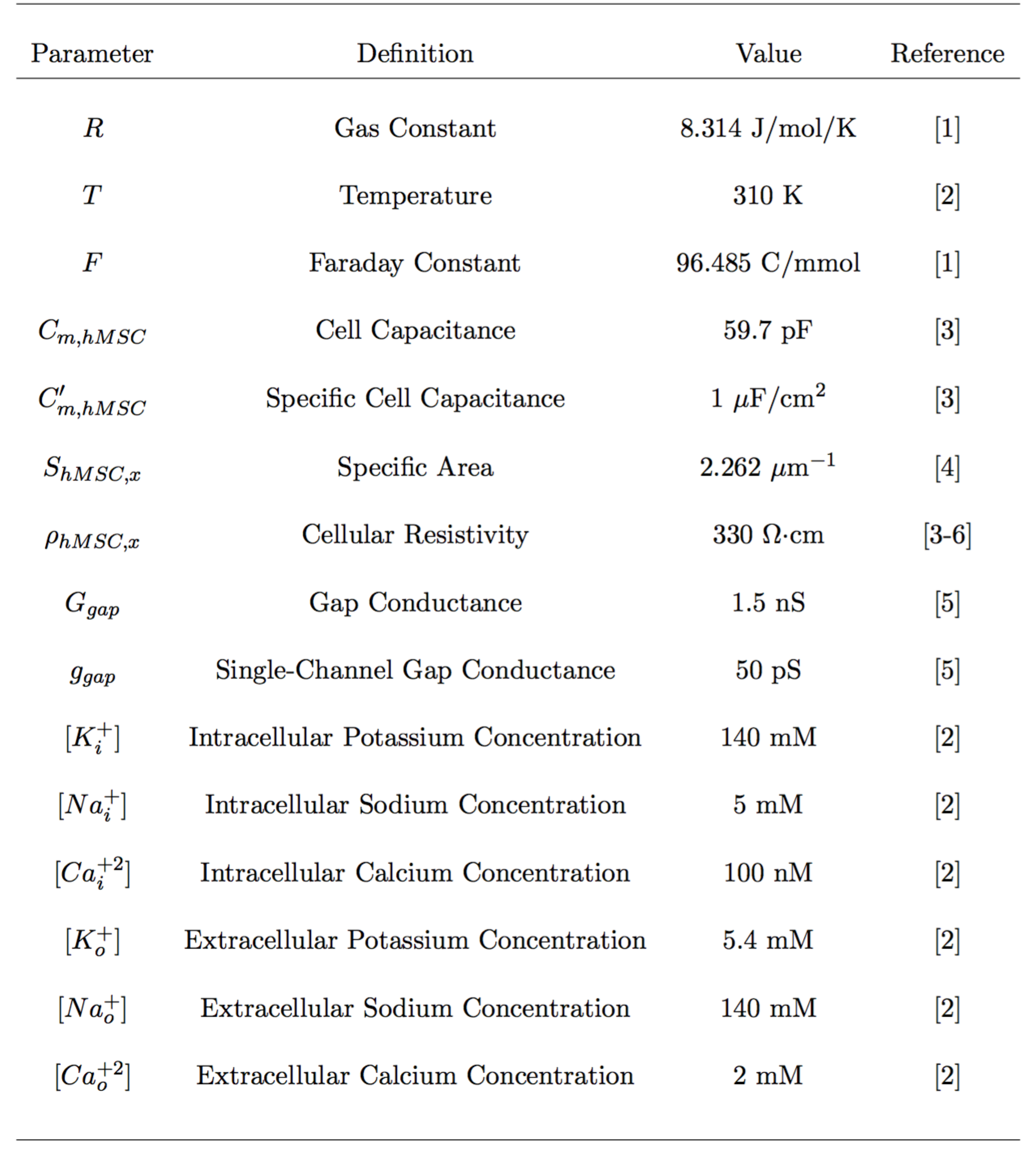


**Table B: IKCa Formulations**

**
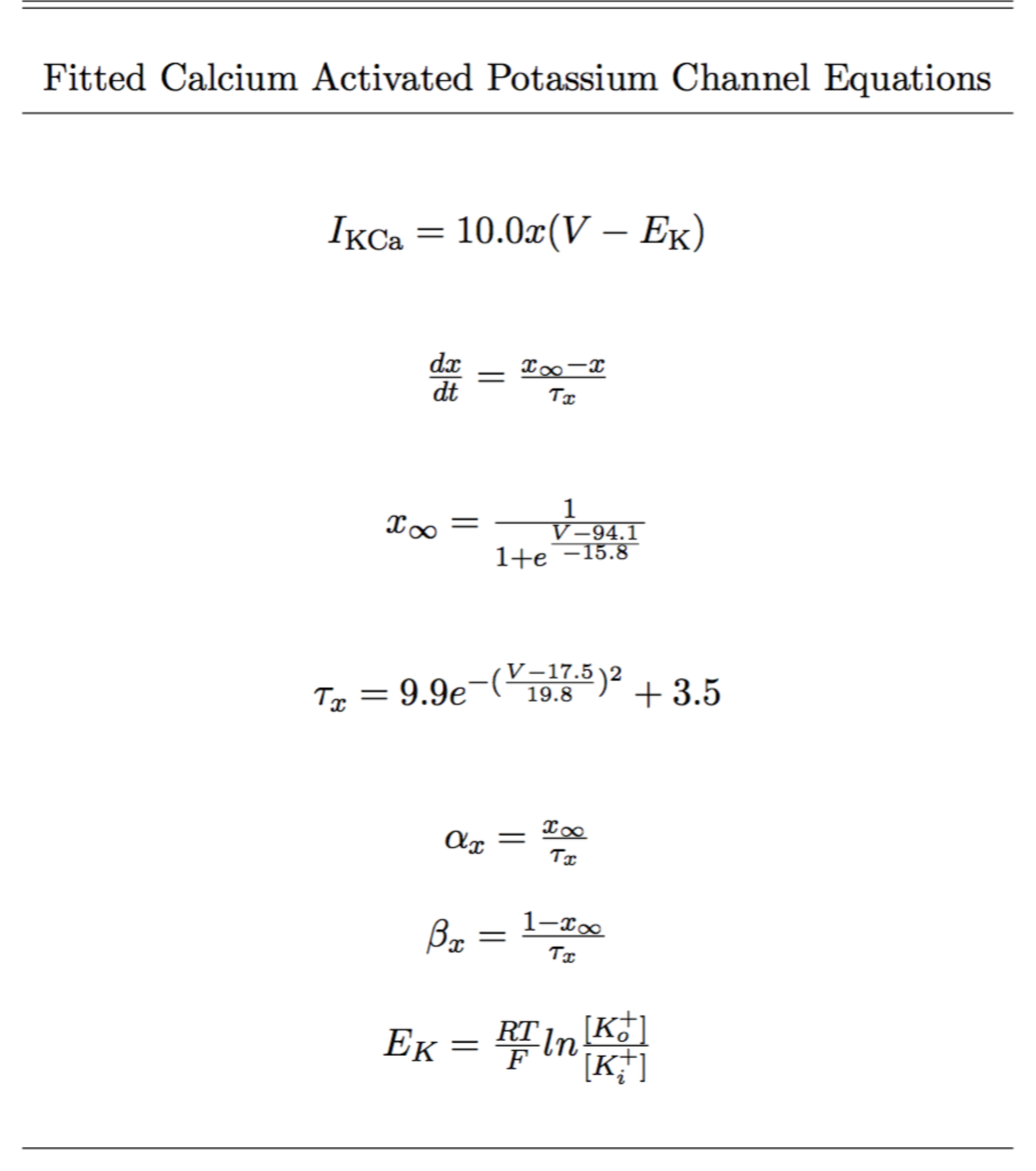
**

**Table C: Idr Formulation**


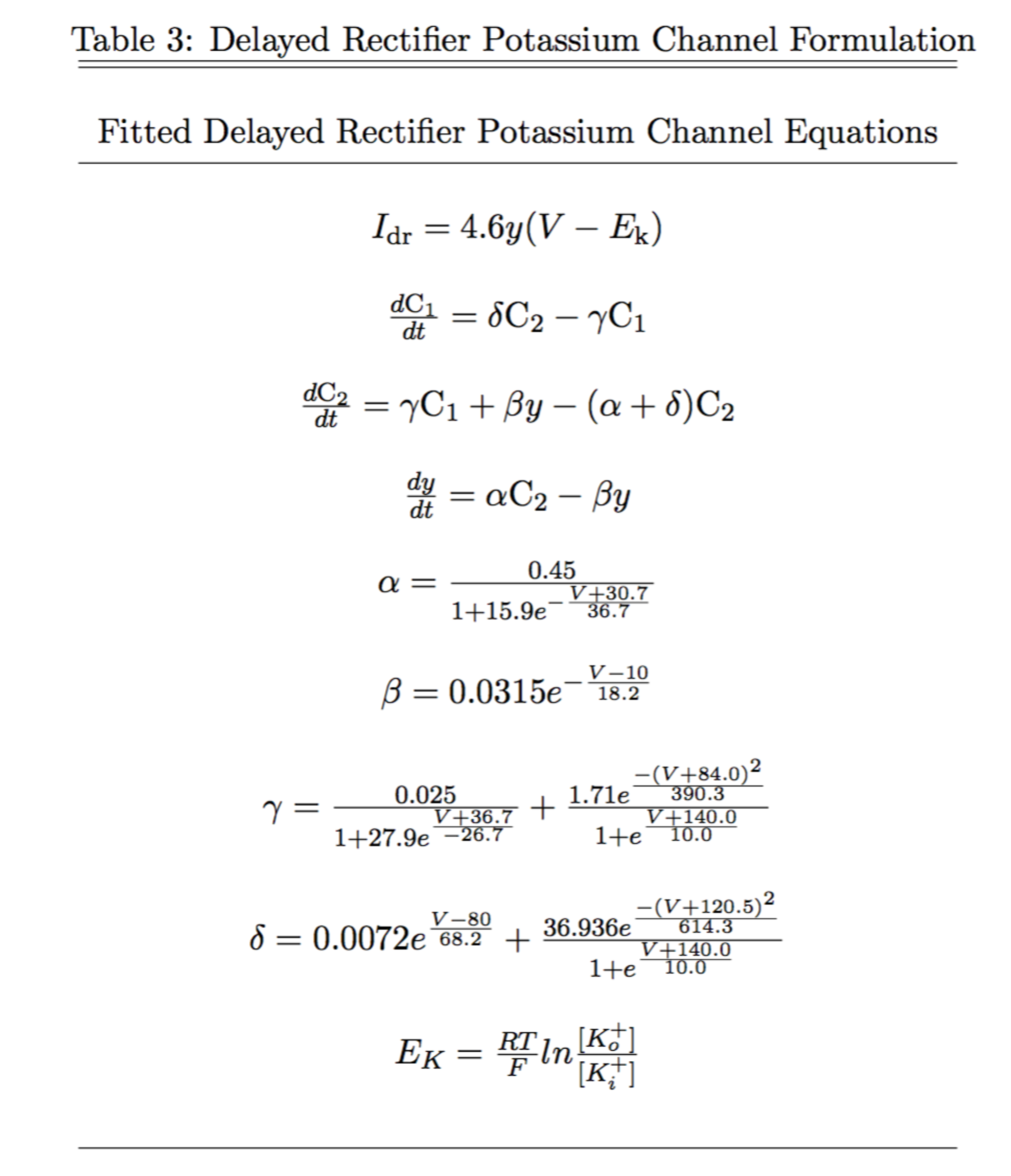


**Table D: ILCa Formulations**


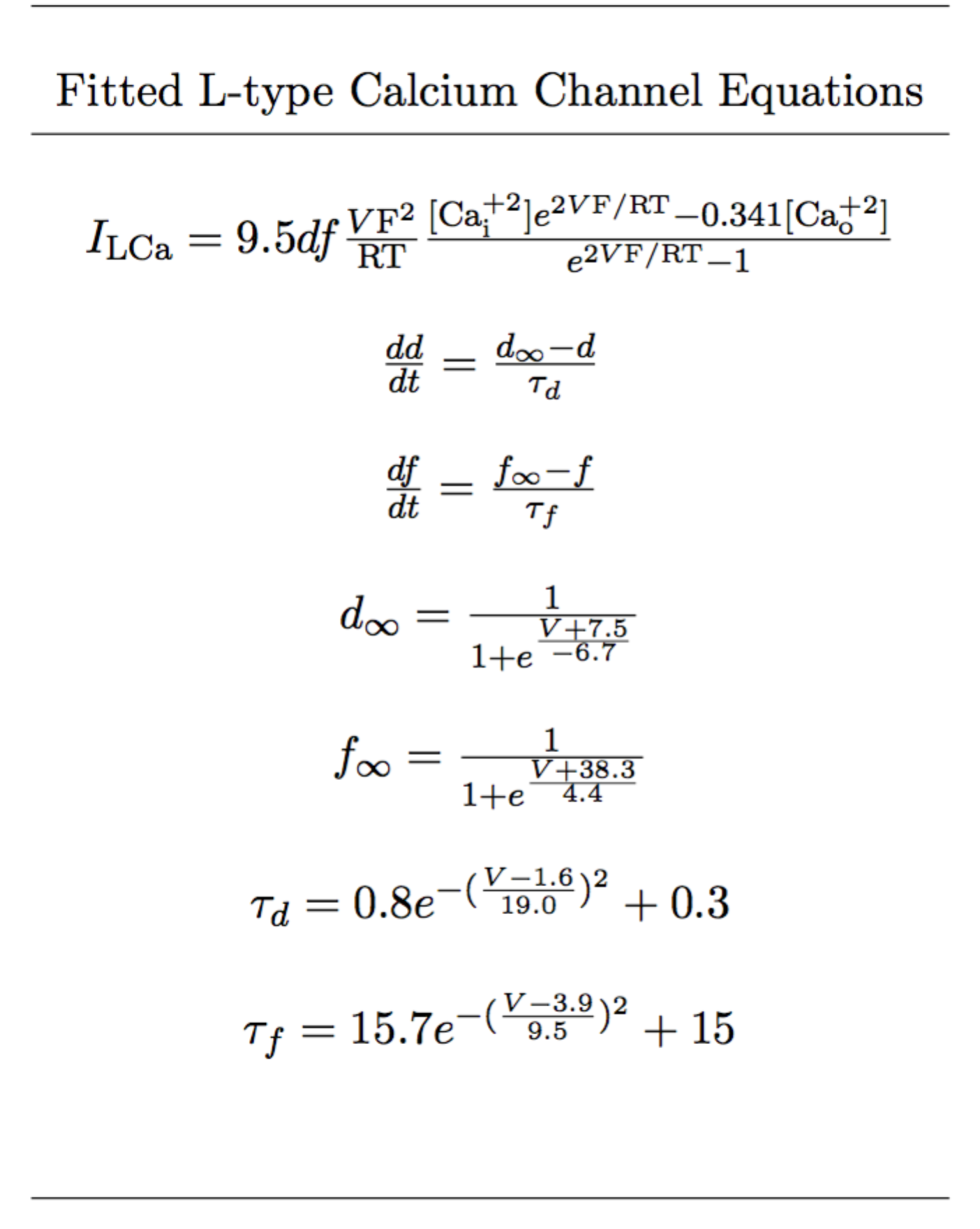


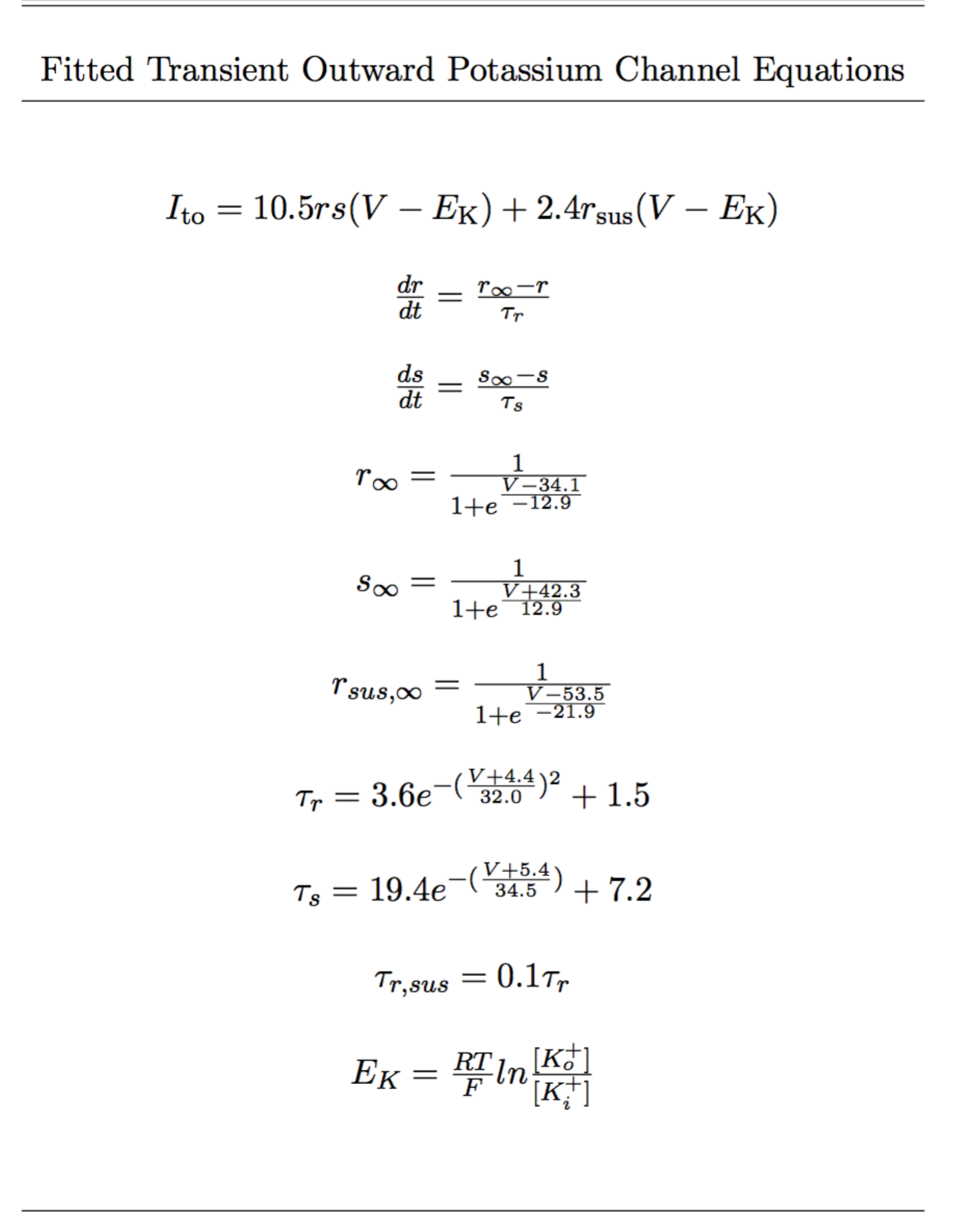
**Table E: Ito Formulations**

**Table F: INa Formulations**


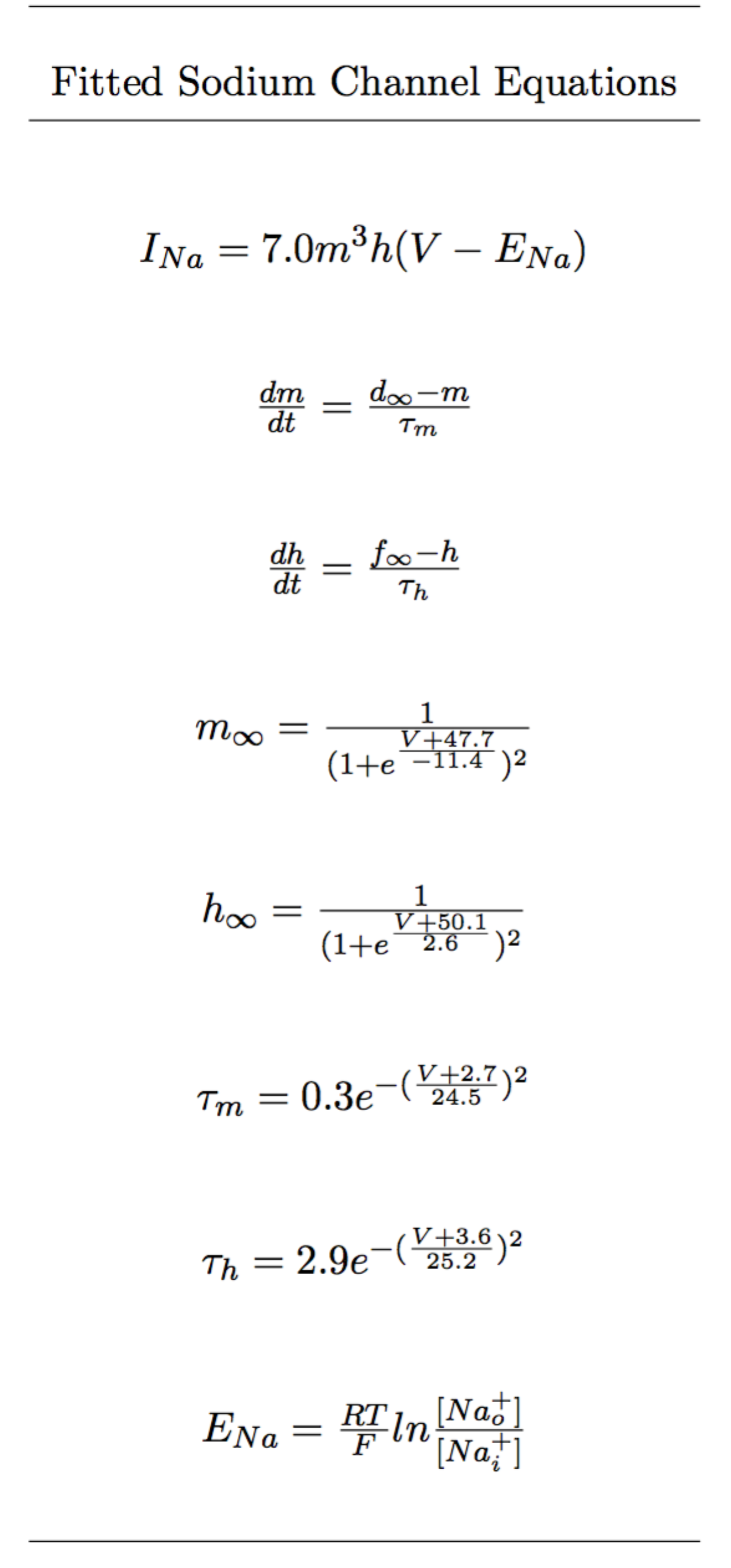


**Table G: IL Formulations**


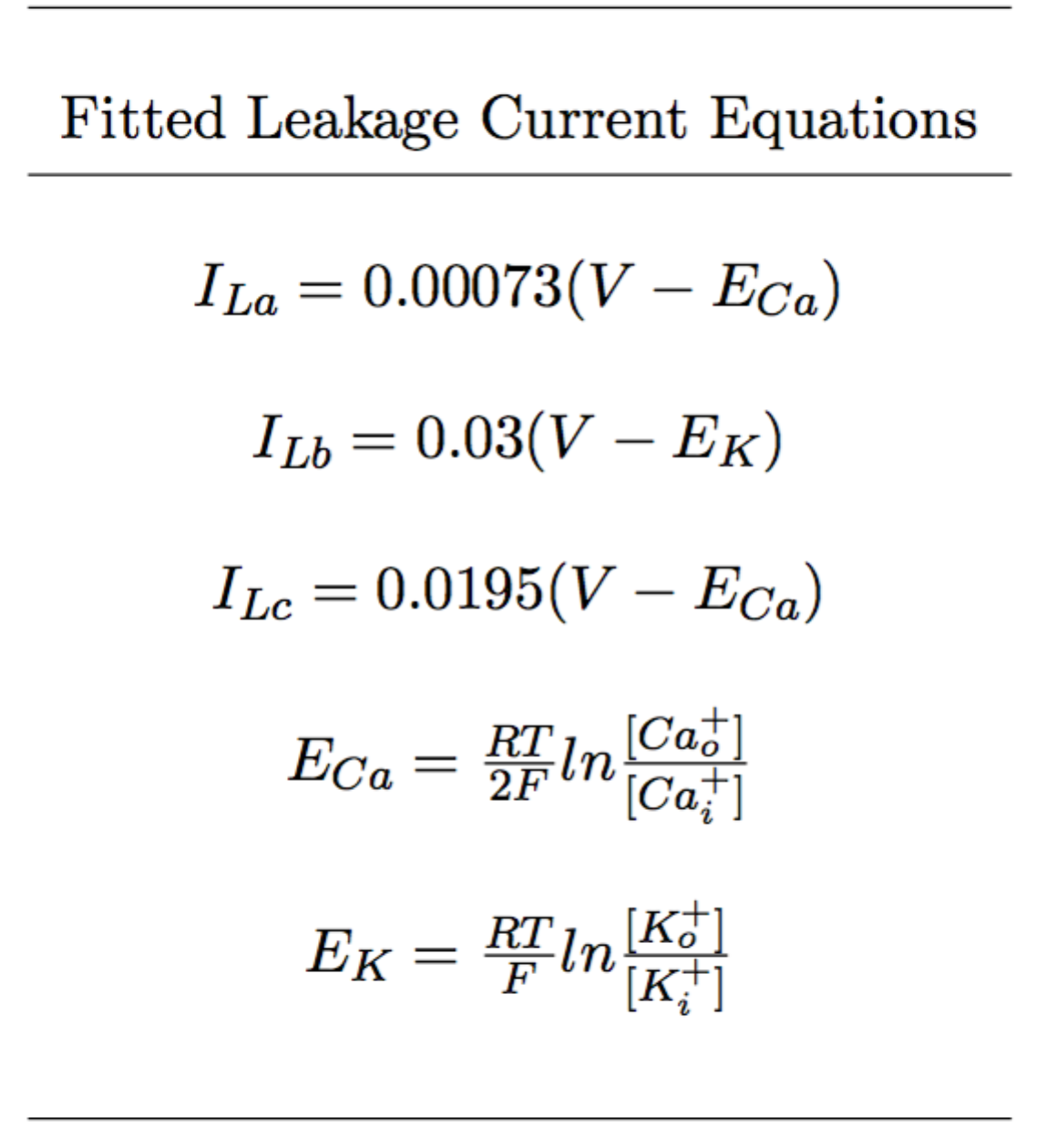


**Table H: Cardiac Sheet Wavelengths with 5%, 15%, and 25% hMSC Insertion**

**Supplementary Tables References:**

[1] Mohr P, Taylor BN, and Newell D. Codata recommended values of the fun- damental physical constants: 2006. Rev. Mod. Phys., 80:633–730, June 2006.

[2] Bard Ermentrout G. Passive cell models: Comp neuroscience.

[3] Li GR, Sun H, Deng X, and Lau CP. Characterization of ionic currents in human mesenchymal stem cells from bone marrow. Stem Cells, 23(3):371–82, March 2005.

[4] Docheva D, Padula D, Popov C, Mutschler W, Clausen-Schaumann H, and Schieker M. Researching into the cellular shape, volume and elasticity of mesenchymal stem cells, osteoblasts and osteosarcoma cells by atomic force microscopy. J. Cell. Mol. Med., 12(2):537–52, April 2008.

[5] Valiunas V, Doronin S, Valiuniene L, Potapova I, Zuckerman J, Walcott B, Robinson RB, Rosen MR, Brink PR, and Cohen IS. Human mesenchymal stem cells make cardiac connexins and form functional gap junctions. J. Physiol., 555(3):617–626, Feb 2004.

[6] Jongsma HJ and Wilders R. Limitations of the dual voltage clamp method in assaying conductance and kinetics of gap junction channels. *Biophysical Journal*, 63(4):942–953, Oct 1992.
