# Supplementary material for: Modeling Electrophysiological Coupling and Fusion between Human Mesenchymal Stem Cells and Cardiomyocytes
Source: PLoS Comput Biol. 2016 Jul 25;12(7):e1005014. doi: 10.1371/journal.pcbi.1005014 (PMC4959759; doi:10.1371/journal.pcbi.1005014)
Supplement: S1 Fig — (DOCX) [file pcbi.1005014.s002.docx]

**S1 Fig: I_KCa_ Steady-State Activation and Time Constant Curves**

**S1 Fig: I_KCa_ Steady-State Activation and Time Constant Curves:** (A) Steady-state activation curve for I_KCa_ together with values derived from mean I-V experimental data [1]. (B) Time constant curve for I_KCa_ together with values derived from experimental voltage clamp data [1].

**References:**

[1] Li GR, Sun H, Deng X, Lau CP. Characterization of ionic currents in human mesenchymal stem cells from bone marrow. Stem cells (Dayton, Ohio). 2005 Mar;23(3):371–382. Available from: http://www.ncbi.nlm.nih.gov/pubmed/15749932.
