# Supplementary material for: Modeling Electrophysiological Coupling and Fusion between Human Mesenchymal Stem Cells and Cardiomyocytes
Source: PLoS Comput Biol. 2016 Jul 25;12(7):e1005014. doi: 10.1371/journal.pcbi.1005014 (PMC4959759; doi:10.1371/journal.pcbi.1005014)
Supplement: S6 Fig — (DOCX) [file pcbi.1005014.s007.docx]

**S6 Fig: Confirming the Resting Membrane Potential of Each hMSC Model**

**S6 Fig: Confirming the Resting Membrane Potential of Each hMSC Model:** Types A (A), B (B), and C (C) hMSCs received a current pulse at t = 0 ms to determine their respective resting membrane potentials. Different leakage conductance and reversal potential values were used for each hMSC to satisfy resting membrane potentials at ~ -35 mV, within the range of empirical data [1].

**References:**

[1] Li GR, Sun H, Deng X, Lau CP. Characterization of ionic currents in human mesenchymal stem cells from bone marrow. Stem cells (Dayton, Ohio). 2005 Mar;23(3):371–382. Available from: http://www.ncbi.nlm.nih.gov/pubmed/15749932.
