# Supplementary material for: Modeling Electrophysiological Coupling and Fusion between Human Mesenchymal Stem Cells and Cardiomyocytes
Source: PLoS Comput Biol. 2016 Jul 25;12(7):e1005014. doi: 10.1371/journal.pcbi.1005014 (PMC4959759; doi:10.1371/journal.pcbi.1005014)
Supplement: S7 Fig — (DOCX) [file pcbi.1005014.s008.docx]

**S7 Fig: Confirming the hMSC-hCM Fusion Model**

**S7 Fig: Confirming the hMSC-hCM Fusion Model:** To validate the method of modeling cell fusion described in this paper, we compared 1:1 hMSC:hCM simulation results for control (G_gap_ = 0 nS, blue line), standard gap conductance (G_gap_ = 1.5 nS, red line), and increasing G_gap_ (15 nS, 150 nS, 1500 nS; yellow, purple, green, respectively) which converge to the case of cell fusion (dashed line) as G_gap_ 🡪 infinity. These two answers converged to the same answers for (A) types A, (B) B, (C) C, and (D) mixed population of hMSCs based on approximate prevalence in vitro [1].

**References:**

[1] Li GR, Sun H, Deng X, Lau CP. Characterization of ionic currents in human mesenchymal stem cells from bone marrow. Stem cells (Dayton, Ohio). 2005 Mar;23(3):371–382. Available from: http://www.ncbi.nlm.nih.gov/pubmed/15749932.
