# Supplementary material for: Modeling Electrophysiological Coupling and Fusion between Human Mesenchymal Stem Cells and Cardiomyocytes
Source: PLoS Comput Biol. 2016 Jul 25;12(7):e1005014. doi: 10.1371/journal.pcbi.1005014 (PMC4959759; doi:10.1371/journal.pcbi.1005014)
Supplement: S8 Fig — (DOCX) [file pcbi.1005014.s009.docx]

**S8 Fig: Configuration of the Heterogeneous Anisotropic hMSC-hCM 2-D Tissue**

**S8 Fig: Configuration of the Heterogeneous Anisotropic hMSC-hCM 2-D Tissue:** A sample 0.5 mm by 0.5 mm section of the monolayer two-dimensional cardiac tissues used in this study. V, I_tot_, and C_m_ represent the voltage, total ionic current, and capacitance used at each node. The diffusion coefficient between hMSCs and their neighboring nodes was D_hCM-hMSC_, while the diffusion coefficient between neighboring hCMs was D_hCM_. To create anisotropic conditions, D_hCM,x_ and D_hCM-hMSC,x_ were four times D_hCM,y_ and D_hCM-hMSC,y_, respectively.
