# Supplementary material for: Modeling Electrophysiological Coupling and Fusion between Human Mesenchymal Stem Cells and Cardiomyocytes
Source: PLoS Comput Biol. 2016 Jul 25;12(7):e1005014. doi: 10.1371/journal.pcbi.1005014 (PMC4959759; doi:10.1371/journal.pcbi.1005014)
Supplement: S9 Fig — (DOCX) [file pcbi.1005014.s010.docx]

**S9 Fig: Underlying Effects of hMSCs on hCM Ionic Currents During an Action Potential**

**S9 Fig: Underlying Effects of hMSCs on hCM Ionic Currents During an Action Potential:** hMSCs were coupled to midcardial hCMs in a 1:1 ratio to better understand the underlying effects of each type of hMSC. The hCM ionic currents analyzed include: (A) I_Na_, (B) I_to_, (C) I_LCa_, (D) I_Ks_, (E) I_Kr_, and (F) I_K1_. The largest effects occurred during phases 2, 3, and 4 of action potentials, when outward potassium currents govern repolarization. hEAG1-functional hMSCs resulted in the earliest initiation of phases 3 and 4.
