# Supplementary material for: Modeling Electrophysiological Coupling and Fusion between Human Mesenchymal Stem Cells and Cardiomyocytes
Source: PLoS Comput Biol. 2016 Jul 25;12(7):e1005014. doi: 10.1371/journal.pcbi.1005014 (PMC4959759; doi:10.1371/journal.pcbi.1005014)
Supplement: S10 Fig — (DOCX) [file pcbi.1005014.s011.docx]

**S10 Fig: Effects of hMSC Coupling and Fusion on Endocardial hCMs**

**S10 Fig: Effects of hMSC Coupling and Fusion on Endocardial hCMs:** The three hMSC models developed in this study were coupled and fused to endocardial hCM electrophysiological models 1:1 to develop insight into how these two cell types interact. (A) Type A hMSCs were coupled and fused to endocardial hCMs, resulting in a significant decrease in endocardial hCM APD. (B) Type B hMSCs were coupled and fused to endocardial hCMs, resulting in a similar effect as with the type A hMSCs. (C) Type C hMSCs, absent of delayed rectifier-like hEAG1 channel activity, had a noticeably smaller effect on endocardial hCM APD. (D) A mixed population of hMSCs (i.e., all three families of hMSCs weighted based on their approximate prevalence in vitro) were coupled and fused to endocardial hCMs, resulting in similar effects as types A and B hMSCs.
