# Supplementary material for: Modeling Electrophysiological Coupling and Fusion between Human Mesenchymal Stem Cells and Cardiomyocytes
Source: PLoS Comput Biol. 2016 Jul 25;12(7):e1005014. doi: 10.1371/journal.pcbi.1005014 (PMC4959759; doi:10.1371/journal.pcbi.1005014)
Supplement: S12 Fig — (DOCX) [file pcbi.1005014.s013.docx]

**S12 Fig: APD Map of Cardiac Tissue With 5% hMSCs Inserted**

**S12 Fig: APD Map of Cardiac Tissue With 5% hMSCs Inserted:** APD maps for cardiac tissue with 5% of types (A) A, (B) B, (C) C, and (D) mixed populations of hMSCs based on approximate prevalence in vitro. Top inset shows the mean APD of the tissue, and bottom left inset shows a 10x zoom on the center of the tissue. White dots represent the random locations of hMSCs.
