# Supplementary material for: Modeling Electrophysiological Coupling and Fusion between Human Mesenchymal Stem Cells and Cardiomyocytes
Source: PLoS Comput Biol. 2016 Jul 25;12(7):e1005014. doi: 10.1371/journal.pcbi.1005014 (PMC4959759; doi:10.1371/journal.pcbi.1005014)
Supplement: S13 Fig — (DOCX) [file pcbi.1005014.s014.docx]

**S13 Fig: Raw APD Restitution Curves Following 5%, 15%, and 25% hMSC-hCM Coupling**

**S13 Fig: Restitution Curves Following 5%, 15%, and 25% hMSC-hCM Coupling:** APD restitution slopes when coupling (A) 5%, (B) 15%, and (C) 25% hMSCs were examined for insight into electrical instability. At 5% hMSC coupling, there were indistinguishable effects on the APD restitution curve. At higher hMSC coupling, each type of hMSC slightly reduced the maximum APD restitution slope compared to the control (0% hMSC) condition.
